# Estimating latent, dynamic processes of breast cancer tumour growth and distant metastatic spread from mammography screening data

**DOI:** 10.1177/09622802211072496

**Published:** 2022-02-01

**Authors:** Alessandro Gasparini, Keith Humphreys

**Affiliations:** Department of Medical Epidemiology and Biostatistics, 27106 Karolinska Institutet, Stockholm, Sweden

**Keywords:** Breast cancer, distant metastatic spread, natural history model, continuous growth model, survival analysis

## Abstract

We propose a framework for jointly modelling tumour size at diagnosis and time to distant metastatic spread, from diagnosis, based on latent dynamic sub-models of growth of the primary tumour and of distant metastatic detection. The framework also includes a sub-model for screening sensitivity as a function of latent tumour size. Our approach connects post-diagnosis events to the natural history of cancer and, once refined, may prove useful for evaluating new interventions, such as personalised screening regimes. We evaluate our model-fitting procedure using Monte Carlo simulation, showing that the estimation algorithm can retrieve the correct model parameters, that key patterns in the data can be captured by the model even with misspecification of some structural assumptions, and that, still, with enough data it should be possible to detect strong misspecifications. Furthermore, we fit our model to observational data from an extension of a case-control study of post-menopausal breast cancer in Sweden, providing model-based estimates of the probability of being free from detected distant metastasis as a function of tumour size, mode of detection (of the primary tumour), and screening history. For women with screen-detected cancer and two previous negative screens, the probabilities of being free from detected distant metastases 5 years after detection and removal of the primary tumour are 0.97, 0.89 and 0.59 for tumours of diameter 5, 15 and 35 mm, respectively. We also study the probability of having latent/dormant metastases at detection of the primary tumour, estimating that 33% of patients in our study had such metastases.

## 1 Introduction

Data collected through cancer screening trials or via population-based studies carried out in the presence of screening programmes enable the estimation of underlying cancer natural history processes. The natural history of breast cancer has traditionally been modelled using either discrete multi-state (semi-) Markov models or continuous tumour growth models, based on using data on tumour characteristics that are collected at diagnosis. Such models have been used to study, e.g., screening effectiveness and over-diagnosis.^1^ The most basic multi-state Markov model, used in this context, is a three-states model (undetectable breast cancer, asymptomatic cancer detectable by screening and symptomatic breast cancer) and another example is the five-states model that sub-divides asymptomatic and symptomatic states by lymph nodes involvement.^2^ Markov models can be easily extended, but model complexity grows quickly. These models have other limitations too, which have been discussed elsewhere.^2^ Biologically-inspired (continuous) tumour growth models are being used increasingly often as an alternative to multi-state Markov models, due to their flexibility and parsimony.^3–10^These models combine a growth model of breast cancer with a continuous function of screening sensitivity (depending on e.g. tumour size or mammographic density) and accommodate random effects to account for individual variations in tumour growth. From fitting such models to observational data it has been shown that Markov models may assume too little individual variation in cancer progression.^6^ These types of continuous growth models have so far been developed only to model data collected up until the time of diagnosis. In this paper, we explore an approach that models events occurring after diagnosis but incorporates the tumour’s natural history. Here, we focus specifically on distant metastatic spread: we describe a framework for modelling tumour characteristics at diagnosis and time to distant metastasis (counted from diagnosis), which is based on specifying a dynamic process of metastatic spread from the onset of the primary tumour. The framework we describe could potentially be extended to other events (such as tumour relapse or death) and can be useful, e.g., for studying personalised screening and treatment effects.

The (metastatic) process of cancer cells disseminating from the primary tumour to a distant organ is complex.^11,12^Arvelo et al.^13^ divide the pathogenesis of metastasis into 10 sequential steps. In the early steps, tumour cells detach from the primary tumour and travel through the vascular system; experimental data show that this first stage is very efficient.^11,12^ For cancer cells to extravasate to a distant organ and to establish metastases they need to then evade multiple mechanisms which are designed to prevent the formation of metastases. Few cells (compared to the large number of cells that shed from the primary tumour) manage to successfully form a metastasis.^14,11^ From observational data, it is not possible to distinguish these two stages; in the current paper, we model successful metastases directly. Finally, in the third stage, newly formed metastases grow until they become clinically detectable.

Over the years, several publications have reported that around 90% of cancer deaths can be attributed to metastatic spread of the primary tumour^15,16^; for some cancers (e.g. brain, heart, liver, airways) local tumours can affect vital organs. In breast cancer, all death will, in essence, be attributable to distant metastases. Patients with breast cancer that initially present with distant metastases are diagnosed with Stage IV disease, which is nearly always incurable. It has also been estimated that among women without detectable metastases at the time of diagnosis (and surgery), and initially diagnosed with early-stage disease, around 30% will ultimately develop metastases months or even years later.^17^ Some of the most common sites of distant metastatic involvement are lung, bone, liver and pleura.^18^ Refined surgical techniques and primary local and systemic therapies are all aimed at preventing metastatic spread and growth.^19^ However, metastatic cells become selected, experience high mutation rates and become resistant to treatments. The growth of these (therapy-resistant) metastases colonies is responsible for the vast majority of cancer-related deaths. Hosseini et al.^20^ have estimated, based on molecular studies, that at least 80% of breast cancer metastases are derived from early disseminated cells (well before diagnosis of the primary tumour) and this had been offered as a reason why early detection and treatment often fail to prevent cancer deaths. A better understanding of the metastatic process is fundamental to understanding approaches aimed at reducing cancer mortality.

This paper is organised as follows. In Section 2, we first describe the modelling framework for tumour growth (upon which we build); we then extend it to incorporate the distant metastatic spread process. A model in the simple setting of no screening is described in Section 2.3, which is then extended to incorporate data collected in the presence of screening (Section 2.4). We show via Monte Carlo simulation that our estimation procedure retrieves the correct model parameters (Section 3). In Section 4, we illustrate our methodology using data from a Swedish population-based case-control study of postmenopausal breast cancer. In Section 5, we assess the robustness of the model (in the absence of screening) to violation of some of the structural assumptions. Finally, we conclude the manuscript with a discussion (Section 6).

## 2 Methods

The model developed in this paper is aimed at being used on breast cancer incident cases, collected in a population of women in which screening is offered. Before dealing with screening, we start by introducing the model in the context of no screening.

### 2.1 Modelling tumour growth

The growth of the primary tumour is assumed to follow an exponential function such that, for a tumour growing according to an inverse growth rate R=r , t years after onset it has volume

(1) $$V(t|r)=V_{\text{cell}}\exp\;(t/r)$$

where $V_{\text{cell}}$ represents an initial volume computed under the assumption that tumours are spherical and have a diameter $d_{\text{cell}}$ = 0.01 mm.

Individual variation in growth rates is accounted for by assuming that inverse growth rates follow a Gamma distribution with shape $\tau_{1}$ and rate $\tau_{2}$ :

(2) $$f_{R}(r)=\frac{\tau_{2}^{\tau_{1}}}{\Gamma (\tau_{1})}r^{\tau_{1}-1}\exp\;(-\tau_{2}r),\;r\geq 0$$

where Γ(⋅) is the Gamma function.

We assume that tumours are detectable with non-zero probability from a given volume $V_{0}$ corresponding to a diameter $d_{0}$ = 0.5 mm. Given the exponential growth model described above, $t_{0}$ represents the time it takes for a single tumour cell to grow until it becomes detectable

$$V_{0}=V_{\text{cell}}\exp\;(t_{0}/r)$$

As in previous work,^3,5,8–10^ we assume that, in the absence of screening, the rate of symptomatic detection at time $T_{\text{det}}=t$ is proportional to the size of the tumour

(3) $$P(T_{\text{det}}\in [t,t+dt)|T_{\text{det}}\geq t,R=r)=\eta V(t,r)dt+o(dt),\;t\geq t_{0}$$

with $P(T_{\text{det}}\in [t,t+dt)|T_{\text{det}}\geq t,R=r)=0\;\forall \;t<t_{0}$ .

For an unscreened population, the density for tumour volume at symptomatic detection can, from the above assumptions, be calculated as follows:

(4) $$f_{V_{\text{det}}}(v_{\text{det}})=\eta \tau_{1}\frac{\tau_{2}^{\tau_{1}}}{(\tau_{2}+\eta (v_{\text{det}}-V_{0}){)}^{\tau_{1}+1}},\;v_{\text{det}}>V_{0}$$

See Plevritis et al.^5^ for more details.

### 2.2 Modelling the metastatic process in the absence of screening

The model for distant metastatic spread is inspired by previous models on localised spread to the lymph nodes.^10^ We will first formulate a model for successful distant metastatic seeding; then, we will formulate a model for time to detectability of each metastasis given detection of the primary tumour. A successful seeding can also be thought of here as a colony that will not be eradicated by treatment at diagnosis. The time to detectability of distant metastases will be related to the growth rate of the primary tumour.

#### 2.2.1 A model for successful distant metastatic seeding

The model for successful distant metastatic seeding assumes that the metastatic process is proportional to the average number of mutations in the cancer cells and the rate of cancer cell division. We use this model form as it has previously been shown to fit well to observed data on the lymph nodes spread. We note however that our methodology can be easily adapted to other models of spread, e.g. one which specifies the rate of spread to be proportional to the current volume of the primary tumour; it can be seen however that such a model would provide a poorer fit to empirical data (than the one we use), as we will later discuss.

Under the model considered here, assuming a constant rate of mutation during cell division, the average number of mutations is proportional to the number of times the tumour cells have divided. Specifically, metastatic seeding is assumed to follow an inhomogeneous Poisson process with intensity function

(5) $$\lambda (t,r)=\sigma^{*}D(t,r{)}^{k}D^{\prime }(t,r)$$

where D(t,r) represents the number of times that cancer cells have divided, and $D^{\prime }(t,r)$ represents the rate of cell division. As explained in Isheden et al.,^10^ the exponent k (with k≥−1 ) adds additional flexibility to the model, implying that metastatic spread can depend on higher powers of tumour mutation or that tumours mutate at an accelerating (or decelerating) rate; this phenomenon is referred to as *genomic instability*.

The number of times that cancer cells have divided can be calculated from the following relation:

(6) $$V_{\text{cell}}\times 2^{D(t,r)}=V(t,r)\Leftrightarrow D(t,r)=\frac{\log\;(V(t,r)/V_{\text{cell}})}{\log\;(2)}$$

It follows that the cumulative intensity function Λ of the Poisson process can be defined as

(7) $$\Lambda (t,r)=\int_{0}^{t}\lambda (u,r)\;du=\sigma {\left[\log\;\left(\frac{\text{V}(\text{t, r})}{{\text{V}}_{\text{cell}}}\right)\right]}^{k+1}$$

where $\sigma =\sigma^{*}/[(k+1)\log\;(2{)}^{k+1}]$ . Using (1), this formula can be re-written as a function of time and inverse growth rates

(8) $$\Lambda (t,r)=\sigma {\left(\frac{t}{r}\right)}^{k+1}$$

The probability of having u successful metastases seeded at detection (time $t_{\text{det}}$ ) can be defined as

(9) $$P(U=u|T_{\text{det}}=t_{\text{det}},R=r)=\frac{\Lambda (t_{\text{det}},r{)}^{u}}{u!}\exp\;\left(-\Lambda (t_{\text{det}},r)\right)$$

and the survival function for the time to the first successful distant metastatic seeding is

(10) $$S(t|R=r)=P(U=0|T_{\text{det}}=t,R=r)=\exp\;\left(-\frac{\sigma }{r^{k+1}}t^{k+1}\right),\;t\geq 0$$

This corresponds to the survival function of a Weibull distribution with scale parameter $\sigma /r^{k+1}$ and shape parameter k+1 . The corresponding hazard function is

(11) $$h(t|R=r)=\frac{\sigma }{r^{k+1}}(k+1)t^{k},\;t\geq 0$$

and the density function is

(12) $$f_{T}(t|R=r)=\frac{\sigma }{r^{k+1}}(k+1)t^{k}\exp\;\left(-\frac{\sigma }{r^{k+1}}t^{k+1}\right),\;t\geq 0$$

We note that (from equation (11)) we can see that, under our modelling assumptions, fast-growing tumours (with low values of r ) have faster rates of distant metastatic spread than slow-growing tumours, which is consistent with observational data (as described in Section 4).

#### 2.2.2 A model for time to first detected distant metastasis

A model for distant metastatic spread needs to be stated in terms of observable quantities (seedings are not observable), such as the presence of distant metastases at diagnosis and the times of distant metastases from diagnosis of the primary tumour. We therefore define a random variable W , which represents the time to diagnosis of the first metastasis counting time from detection of the primary tumour. Moreover, we introduce the following assumptions:

1. The time from metastatic seeding to detection of a given metastasis takes the individual-specific time $t_{0}$ , i.e. the same time that the primary tumour requires to grow from a single cell to a volume of $V_{0}$ .
2. We assume that breast cancers are only diagnosed through the primary tumour, and not by the clinical manifestation of metastases.
3. We make an exception to the above for metastases growing to a detectable size before the diagnosis of the primary tumour: these metastases will be visible at diagnosis of the primary tumour, but not detected beforehand.

**Figure 1.**
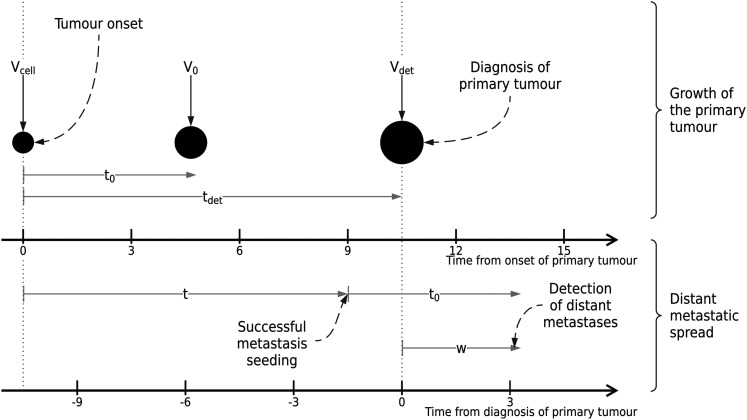
Illustration of key time points for the proposed modelling framework, including tumour volumes at each relevant point in time. Two distinct time scales are included to illustrate the models introduced in Sections 2.2.1 and 2.2.2.

For an individual with a tumour that reaches size $V_{0}$ after $t_{0}$ years from onset, who experiences their first metastatic seeding at time t , and has symptomatic detection of the primary tumour at time $t_{\text{det}}$ , W takes the value $w=t+t_{0}-t_{\text{det}}$ . Figure 1 summarises the key time points (on the different time scales) and corresponding tumour volumes. If the tumour grows with inverse growth rate r , then we can write $t_{\text{det}}=r\log\;(v_{\text{det}}/V_{\text{cell}})$ and $t_{0}=r\log\;(V_{0}/V_{\text{cell}})$ , where $v_{\text{det}}$ is the volume of the primary tumour at symptomatic detection. This allows re-writing the density of W as

(13) $$f_{W}(w|V_{\text{det}}=v_{\text{det}},R=r)=\frac{\sigma }{r}\left(k+1\right){\left(\frac{w}{r}+\log\;\frac{v_{\text{det}}}{V_{0}}\right)}^{k}\exp\;\left[-\sigma {\left(\frac{w}{r}+\log\;\frac{v_{\text{det}}}{V_{0}}\right)}^{k+1}\right]$$

where $V_{\text{det}}$ is a random variable representing volume of the tumour at detection. The survival and hazard functions follow as

(14) $$S(w|V_{\text{det}}=v_{\text{det}},R=r)=\exp\;\left[-\sigma {\left(\frac{w}{r}+\log\;\frac{v_{\text{det}}}{V_{0}}\right)}^{k+1}\right]$$

and

(15) $$h(w|V_{\text{det}}=v_{\text{det}},R=r)=\frac{\sigma }{r}\left(k+1\right){\left(\frac{w}{r}+\log\;\frac{v_{\text{det}}}{V_{0}}\right)}^{k}$$

Equations (13) to (15) are defined for $w\geq r\log\;(V_{0}/v_{\text{det}})$ , with the bound corresponding to the extreme case of a metastasis being seeded at the same time that the primary tumour originated. Importantly, the formulae above are now expressed in terms of tumour sizes at diagnosis (i.e. $v_{\text{det}}$ ), which are directly observable.

We now introduce additional assumptions: we assume that the metastatic seeding completely stops at diagnosis of the primary tumour and that already seeded, successful colonies are not affected. These assumptions constrain the density and hazard functions – (13) and (15) – to be null for values of $w>t_{0}$ , or equivalently, for $w>r\log\;(V_{0}/V_{\text{cell}})$ . As a result (inverse growth rate-specific) survival functions (for fixed values of $v_{\text{det}}$ ) will be constant beyond time $w=r\log\;(V_{0}/V_{\text{cell}})$ after diagnosis, that is

(16) $$S(w|V_{\text{det}}=v_{\text{det}},R=r)=\left\{ \begin{array}{cc} \exp\;\left[-\sigma {\left(\frac{w}{r}+\log\;\frac{v_{\text{det}}}{V_{0}}\right)}^{k+1}\right] & \text{if}\;0\leq w\leq r\log\;(V_{0}/V_{\text{cell}})\\ \exp\;\left[-\sigma {\left(\log\;\frac{v_{\text{det}}}{V_{\text{cell}}}\right)}^{k+1}\right] & \text{if}\;w>r\log\;(V_{0}/V_{\text{cell}}) \end{array}\right.$$

These additional assumptions (together with those introduced earlier) are used to formulate the joint probability of tumour size at diagnosis and time to the first detection of distant metastasis, as described in the following section. We summarise the complete set of assumptions in Table 1. Alternative, less stringent assumptions concerning the spread and growth of distant metastases, as well as treatment effects, could in principle be incorporated, as we discuss later. 

**Table 1.** A summary of the model components, parameters and assumptions for the joint model of tumour growth, detection and distant metastatic spread.

Functional forms	Parameters and Coefficients	Comments
*Model for growth of the primary tumour:*		
Exponential growth from single cell of volume $V_{\text{cell}}$ (equation (1)).	$V_{\text{cell}}$	$V_{\text{cell}}$ is the volume of a sphere with a diameter $d_{\text{cell}}$ of 0.01 mm.
Inverse growth rates, r , follow a Gamma distribution.	$\tau_{1},\tau_{2}>0$	
Model for symptomatic detection of the primary tumour^*^:		
The rate of symptomatic detection in the absence of screening is proportional to tumour volume (equation (3)).	η	
Model for screen detection of the primary tumour^*^:		
Screening sensitivity follows a logistic function (equation (17)).	$\beta_{0},\beta_{1}\ldots ,\beta_{k}\in R$	In principle, any covariate can be included
*Model for distant metastatic spread:*		
Density, hazard, and survival functions for distant metastatic seeding are described in equations (13) to (16). Metastatic seeding occurs as an inhomogeneous Poisson process with rate proportional to number of cell divisions and rate of growth of the primary tumour. Seeding is assumed to fully stop after diagnosis of the primary.	σ>0,k>−1	Based on Isheden et al.^10^ we fix k=4 , but k can be estimated from data.
Model for detection of distant metastases^*^:		
Metastases are detected at a time $t_{0}$ after seeding at the distant site, where $t_{0}$ is the time the primary tumour takes to grow to volume $V_{0}$ . Detection of metastases prior to detection of the primary tumour is delayed until diagnosis (of the primary).	$V_{0}$	$V_{0}$ is assumed to correspond to a diameter $d_{0}$ of 0.5 mm.

* Detection of the primary tumour is assumed to be independent of distant metastases: cancers are always detected via the primary tumour.

### 2.3 Likelihood function in the absence of screening

We describe the joint likelihood of tumour size and time to first distant metastasis, for a sample of incident cases from a stable disease population.^9^ Three types of observations contribute to the likelihood function:

1. Observed events, i.e. metastases that are diagnosed after diagnosis of the primary tumour ( w≥0 ) contribute to the likelihood with density
$$\begin{array}{rl} \;f_{W,V_{\text{det}}}(w,v_{\text{det}}) & =f_{W}(w|V=v_{\text{det}})f_{V_{\text{det}}}(v_{\text{det}})\\ & =f_{V_{\text{det}}}(v_{\text{det}})\int_{R}f_{W}(w|V=v_{\text{det}},R=r)f_{R}(r|V_{\text{det}}=v_{\text{det}})\;dr\\ & =f_{V_{\text{det}}}(v_{\text{det}})\int_{w/\log\;\frac{V_{0}}{V_{\text{cell}}}}^{+\infty }f_{W}(w|V=v_{\text{det}},R=r)f_{R}(r|V_{\text{det}}=v_{\text{det}})\;dr \end{array}$$
  where $f_{W}(\cdot )$ corresponds to equation (13). We note that if events were observable before diagnosis (which, under assumption 3 in Section 2.2.2, they are not), the domain of R would have a subject-specific lower limit of $r\geq w\log\;(V_{0}/v_{\text{det}})$ .
2. Left censored observations, i.e. individuals with detected metastases at the time of diagnosis ( w<0 ). These observations will contribute to the likelihood with their probability of having at least one successful metastasis, which can be derived as
$$f_{V_{\text{det}}}(v_{\text{det}})\left\{1-\exp\;\left[-\sigma {\left(\log\;\frac{v_{\text{det}}}{V_{0}}\right)}^{k+1}\right]\right\}$$
  We note that this quantity is independent of the inverse growth rate r .
3. Right censored observations, i.e. individuals that do not develop metastases before the end of follow-up. These observations will contribute with the survival probability introduced in equation (16), that is
$$\begin{array}{rl} \;f_{V_{\text{det}}} & (v_{\text{det}})\{\int_{w/\log\;\frac{V_{0}}{V_{\text{cell}}}}^{+\infty }\exp\;\left[-\sigma {\left(\frac{w}{r}+\log\;\frac{v_{\text{det}}}{V_{0}}\right)}^{k+1}\right]f_{R}(r|V_{\text{det}}=v_{\text{det}})\;dr\\ & \;+\int_{0}^{w/\log\;\frac{V_{0}}{V_{\text{cell}}}}\exp\;\left[-\sigma {\left(\log\;\frac{v_{\text{det}}}{V_{\text{cell}}}\right)}^{k+1}\right]f_{R}(r|V_{\text{det}}=v_{\text{det}})\;dr\}=\\ f_{V_{\text{det}}} & (v_{\text{det}})\{\int_{w/\log\;\frac{V_{0}}{V_{\text{cell}}}}^{+\infty }\exp\;\left[-\sigma {\left(\frac{w}{r}+\log\;\frac{v_{\text{det}}}{V_{0}}\right)}^{k+1}\right]f_{R}(r|V_{\text{det}}=v_{\text{det}})\;dr\\ & \;+\exp\;\left[-\sigma {\left(\log\;\frac{v_{\text{det}}}{V_{\text{cell}}}\right)}^{k+1}\right]F_{R}(w/\log\;(V_{0}/V_{\text{cell}})|V_{\text{det}}=v_{\text{det}})\} \end{array}$$

Under our modelling assumptions, the density for the inverse growth rates, conditional on volume (which appears in the likelihood contribution for observed events and right-censored observations), can be written^9^ as

$$\begin{array}{rl} \;f_{R} & \left(r|V_{\text{det}}=v_{\text{det}})\;=\frac{\left[\tau_{2}+\eta (v_{\text{det}}-V_{0})\right]}{\Gamma (\tau_{1}+1)}\{r[\tau_{2}+\eta (v_{\text{det}}-V_{0})]{\}}^{(\tau_{1}+1)-1}\exp\;\{-r[\tau_{2}+\eta (v_{\text{det}}-V_{0})]\right\} \end{array}$$

and has corresponding cumulative distribution function

$$F_{R}(r|V_{\text{det}}=v_{\text{det}})=\frac{\gamma (\tau_{1}+1,r[\tau_{2}+\eta (v_{\text{det}}-V_{0})])}{\Gamma (\tau_{1}+1)}$$

where γ(⋅) and Γ(⋅) are the lower incomplete Gamma function and the Gamma function, respectively.

To calculate the likelihood we need to approximate the integrals appearing in the three quantities described above (which do not have a closed-form); to do this we use numerical integration. Once the full likelihood function is formulated, it can be maximised using any general-purpose optimiser such as optim in R.^21^Standard errors for the model parameters can be obtained using, e.g. the observed information matrix at the optimum, or the bootstrap. Using Monte Carlo simulation, we show, in the supplementary material available online, that we can maximise the likelihood function of this model and retrieve the correct model parameters when simulating from a cohort in the absence of screening. We note that the simulation was carried out treating tumour size as a continuous variable; for reasons described later, when we implement our procedure for data collected in the presence of screening (in Section 2.4), we discretise tumour size.

We note again that this likelihood function is valid for samples of incident cases under *stable disease* assumptions: specifically, that (1) the rate of births in the population, (2) the distribution of age at tumour onset, and (3) the distribution of time to symptomatic detection are constant across calendar years. Stable disease assumptions are discussed in more detail in Isheden and Humphreys.^9^

### 2.4 Likelihood function for a screened population

So far, we have described how to estimate our model for samples of cases that are detected symptomatically in the absence of screening: we now extend our approach to allow the analysis of data from incident cases collected in the presence of screening. In Sweden, where our study is based (Section 4), mammography screening was introduced in 1974, and nationwide coverage was achieved in 1997. Nowadays, all women in Sweden between the ages of 40 and 74 years old are called to screening every 2 years (or one and a half years, depending on the country of residence).

First, we specify a model for screening sensitivity. We assume that the probability of a positive screening test, given the presence of a tumour in the breast, follows a logistic function:

(17) $$\mathrm{Screening}\;\mathrm{sensitivity}=\frac{\exp\;(\beta_{1}+\beta_{2}d)}{1+\exp\;(\beta_{1}+\beta_{2}d)},\;d\geq 0.5\;\text{mm}$$

where d is the diameter of the tumour. We note that other covariates (such as mammographic density) can easily be included in this model by extending the linear predictor. We assume that screening sensitivity is zero for d<0.5 mm, that is, tumours that are smaller than 0.5 mm (in diameter) are not detectable via screening. The screening sensitivity function is used (alongside the other sub-models introduced in the previous sections) to formulate the likelihood function in a screened population, as we now describe.

The contribution to the likelihood function is formulated as a conditional joint probability: the joint probability of tumour size and the distant metastatic outcome, given mode of detection (screen-detected versus symptomatic) and the history of previous (negative) screens. We rely on stable disease assumptions to formulate the likelihood, and we use a procedure (and notation) similar to that described in Isheden and Humphreys that was developed for modelling tumour size with data collected in the presence of screening.^9^

-
A is used to denote that there is an (as of yet undetected) tumour in a woman’s breast at a specified point in time.
-
$B_{0}$ is used to denote that a tumour is screen-detected.
-
$B^{c}=B_{1}^{c}\cap B_{2}^{c}\cap \cdots \cap B_{p}^{c}$ is used to denote a series of time-ordered p negative screens, taken prior to a tumour being detected.

#### 2.4.1 Likelihood function for screen-detected tumours

The likelihood contribution for screen-detected (*SD*) tumours can be written as

$$L_{v,w}^{\text{SD}}\propto P(B_{0}|V=v)P(V=v,W=w|A)P(B^{c}|A,V=v,W=w)$$

where V is a random variable representing the tumour volume at the time of screen detection – but can equally be thought of as being at an arbitrary point in time; v is the observed size of the tumour at screen-detection. W is the distant metastatic outcome, written as a continuous random variable for now; later, we will accommodate left-censored observations, observed events, and right-censored observations). The term representing *screening history*, i.e. $P(B^{c}|\cdot )$ , can be omitted for individuals with no history of previous negative screens.

The joint probability of tumour size and metastasis can be re-written as

P(V=v,W=w|A)=P(W=w|V=v)P(V=v|A)

since W is independent of A when conditioning on V . Using Theorem 2 in Isheden and Humphreys,^9^ we can re-write P(V=v,W=w|A) as

$$P(V=v,W=w|A)\propto P(W=w|V=v)P(V_{\text{det}}=v)$$

where $P(V_{\text{det}})$ is defined from equation (4).

The screening history term in $L_{v,w}^{\text{SD}}$ can be re-written as

$$P(B^{c}|A,V=v,W=w)=\int_{R}\left[\prod_{q=1}^{p}P(B_{q}^{c}|R=r,V=v)\right]f_{R}(r|V=v,W=w,A)\;dr$$

Now, the conditional density of growth rates can be re-written as

$$f_{R}(r|V=v,W=w,A)=\frac{\;f_{W}(w|R=r,V=v)f_{R}(r|V=v,A)}{f_{W}(w|V=v)}$$

and according to Theorem 3 in Isheden and Humphreys,^9^ we can write

$$f_{R}(r|V=v,A)=f_{R}(r|V_{\text{det}}=v)$$

Therefore, $L_{v,w}^{\text{SD}}$ can be formulated as

(18) $$\begin{array}{c} L_{v,w}^{\text{SD}}\propto P(B_{0}|V=v)\times P(V_{\text{det}}=v)\times \int_{R}\left[\prod_{q=1}^{p}P(B_{q}^{c}|R=r,V=v)\right]f_{W}(w|R=r,V=v)f_{R}(r|V_{\text{det}}=v)\;dr \end{array}$$

The likelihood contributions differ between observed events, left-censored, and right-censored observations, but in all cases, they will follow the form of (18).

For screen-detected individuals with observed events

(19) $$\begin{array}{c} L_{v,w}^{\text{SD}}\propto P(B_{0}|V=v)\times P(V_{\text{det}}=v)\times \int_{w_{i}/\log\;\frac{V_{0}}{V_{\text{cell}}}}^{+\infty }\left[\prod_{q=1}^{p}P(B_{q}^{c}|R=r,V=v)\right]f_{W}(w|R=r,V=v)f_{R}(r|V_{\text{det}}=v)\;dr \end{array}$$

where the first term corresponds to the screening sensitivity (17), $f_{W}(w|R=r,V=v)$ is identical to the right-hand-side of (13), with $v_{\text{det}}$ replaced by v , and $f_{R}(r|V_{\text{det}}=v)$ is obtained similarly from the conditional inverse growth rate distribution defined in Section 2.3.

The likelihood contribution of screen-detected individuals that are left censored ( w<0 ) can be written as

(20) $$\begin{array}{cc} L_{v,w}^{\text{SD}} & \propto P(B_{0}|V=v)\times P(V_{\text{det}}=v)\times \left\{1-\exp\;\left[-\sigma {\left(\log\;\frac{v}{V_{0}}\right)}^{k+1}\right]\right\}\\ & \;\times \int_{0}^{+\infty }\left[\prod_{q}P(B^{c}|R=r,V=v)\right]\times f_{R}(r|V_{\text{det}}=v)\;dr \end{array}$$

and the likelihood contribution from right censored, screen-detected individuals can be written as

(21) $$\begin{array}{cc} L_{v,w}^{\text{SD}} & \propto \;P(B_{0}|V=v)\times P(V_{\text{det}}=v)\times \{\int_{w_{i}/\log\;\frac{V_{0}}{V_{\text{cell}}}}^{+\infty }\left[\prod_{q}P(B^{c}|R=r,V=v)\right]\times \exp\;\left[-\sigma {\left(\frac{w_{i}}{r}+\log\;\frac{v}{V_{0}}\right)}^{k+1}\right]\\ & \times f_{R}(r|V_{\text{det}}=v)\;dr+\int_{0}^{w_{i}/\log\;\frac{V_{0}}{V_{\text{cell}}}}\left[\prod_{q}P(B^{c}|R=r,V=v)\right]\times \exp\;\left[-\sigma {\left(\log\;\frac{v}{V_{\text{cell}}}\right)}^{k+1}\right]\times f_{R}(r|V_{\text{det}}=v)\;dr\} \end{array}$$

Because $L_{v,w}^{\text{SD}}$ is known only up to a proportionality constant, when evaluating the likelihood in practice, we discretise tumour size in small intervals. The density tumour volume $f_{V_{\text{det}}}(v_{\text{det}})$ is then approximated by the probability of being in a given interval size $I_{i}$ :

$$f_{V_{\text{det}}}(v_{\text{det}})\approx P(V_{\text{det}}\in I_{i})$$

We assume the following diameter intervals (in mm): [0.5,1.5) , [1.5,2.5) , [2.5,7.5) , [7.5,12.5) … , [67.5,72.5) , [72.5,85) , [85,95) … , [145,155) , as in previous work.^9,10^ The second term in (19) to (21) is then approximated by the probability of being in a given interval size $I_{i}$ . The first term within each of the integrals in (19) to (21) is the (conditional) probability of p negative screens, which can be calculated by backwards projection: in brief, these terms are calculated by summing over all sizes up to and including the tumour size at diagnosis, weighting according to their conditional probabilities at the previous screen(s), and calculating each $P(B_{q}^{c}|\cdot )$ by projecting to tumour sizes at earlier screens based on the midpoints of size intervals at the positive screen and the most recent negative screen. The backwards projection procedure is described in more detail elsewhere.^8–10^ When marginalising over the inverse growth rate distribution in each case, we respect the same integration bounds for growth rates as in Section 2.3. Discretising time makes it straightforward to calculate the likelihood when it is known only to a proportionality constant, without being too computationally demanding. For example, for individuals with metastasis detection time right censored at W=w , we have to evaluate both the right-hand-side of (21), as it is, and the same quantity, but with 1−exp(⋅) terms in place of exp(⋅) terms, for all tumour size intervals.

#### 2.4.2 Likelihood function for tumours detected symptomatically

For symptomatic (*SYM*) cases with a history of negative screens, the likelihood can be written as

$$L_{v,w}^{\text{SYM}}\propto P(V_{\text{det}}=v_{\text{det}},W=w)P(B^{c}|V_{\text{det}}=v_{\text{det}},W=w)$$

For individuals with no history of previous negative screens, the term for *screening history* $P(B^{c}|\cdot )$ is omitted.

The contributions to the likelihood for individuals that are detected symptomatically are evaluated analogously to (19) to (21), with the first term, i.e. the screening sensitivity $P(B_{0}|V_{\text{det}}=v_{\text{det}})$ , omitted.

#### 2.4.3 Evaluation of the likelihood

The likelihood functions described in Sections 2.4.2 and 2.4.3 contain integrals that have no closed form. We approximate them numerically using a procedure similar to that described in Section 2.3 in the absence of screening. Once the full likelihood function is formulated, it can, again, be maximised using any general-purpose optimiser with standard errors calculated using, e.g. the observed information matrix at the optimum or the bootstrap.

### 2.5 Model-based estimation of the probability of distant metastasis

The vast majority of breast cancer patients do not have a detected distant metastasis at diagnosis: for these, we can estimate conditional survival probabilities using the model described in Section 2.4. These model-based predictions still rely on assumptions that were previously outlined in Table 1; the calculations implicitly assume removal of the primary tumour at the time of diagnosis, which fully stops the process of metastatic seeding.

For example, for symptomatically-detected tumours, survival probability predictions at a given time $w^{*}$ can be defined for subjects that are event-free at time of detection as

$$P(W>w^{*}|V=v,W>0,B^{c})=\frac{P(W>w^{*}|V=v,B^{c})}{P(W>0|V=v)}$$

The numerator is proportional to

$$\int_{R}\left[\prod_{q}P(B^{c}|R=r,V=v)\right]\times S(w|V=v,R=r)\times f_{R}(r|V_{\text{det}}=v)\;dr$$

where S(w|⋅) is defined in (16). More precisely, i.e. writing the integration over the specific domain of R , this is equal to the terms within curly brackets on the right-hand side of equation (21). To evaluate the numerator (i.e. not just up to a proportionality constant) we need to calculate such terms, first based on using S(w|⋅) (as in equation (21)) and then by substituting S(w|⋅) for [1−S(w|⋅)] . That is, we deal with the normalisation in a similar way to that described for the likelihood evaluation.

The denominator is calculated as the probability of having no detected metastases at detection of the primary tumour, which is independent of r , and thus also of $B^{c}$ :

$$P(W>0|V=v)=\exp\;\left[-\sigma {\left(\log\;\frac{v}{V_{0}}\right)}^{k+1}\right]$$

For screen-detected cancers, the survival probabilities are calculated in exactly the same way (since $P(B_{0}|V=v)$ terms in the numerator and denominator cancel out). Differences between screen- and symptomatically detected cancers get captured by screening histories – symptomatically detected cancers will be more likely than screen-detected cancers to have recent negative screens and therefore to have higher probabilities of fast-growing tumours and higher rates of metastases (see results in Section 4).

## 3 Simulation study

### 3.1 Aim

We designed a simulation study aimed at assessing the ability to retrieve the true data-generating parameters of the model for data collected in the presence of screening, i.e. the model described in Section 2.4. In other words, we aim to test that our implementation can correctly estimate parameter values when the model is correctly specified.

### 3.2 Data-generating mechanisms

We simulated independent datasets, each with 1500 individuals. We assumed that tumours originate from a single spherical cell of volume $V_{\text{cell}}$ corresponding to a diameter of $d_{\text{cell}}$ = 0.01 mm; as described earlier, we assumed that tumours are detectable only once they have reached a given volume $V_{0}$ corresponding to a diameter $d_{0}$ = 0.5  mm. We assumed the parameter k to be fixed at k=4 .

We simulated inverse growth rates from a Gamma distribution with shape $\tau_{1}=2.5$ and rate $\tau_{2}=4.5$ . This corresponds to a Gamma distribution with a mean of 0.56 and a variance of 0.12 . These parameter values, as well as others (below), were chosen based on previous experience of fitting tumour growth models to observational data.^8–10^ Next, we simulated the volumes of the tumours at symptomatic detection based on equation (4), fixing the parameter $\eta^{\prime }=-\log\;(\eta )=8.5$ .

We simulated age at onset of breast cancer with age-increasing rates based on a procedure described previously,^8–10^ which makes use of published breast cancer incidence rates.^22^ We enforced a screening programme starting at the age of 40 years old, with screens every 2 years. We did not include death in our simulations; deaths (due to causes other than breast cancer) would introduce minimal biases in estimates of growth rate distributions, see Isheden et al.^10^ Tumours were detected via screening with probabilities corresponding to equation (17), assuming $\beta_{1}=-5$ and $\beta_{2}=0.7$ .

We simulated the timing of the first metastatic seeding using the inversion method, as described by Bender et al.^23^; we assumed σ=exp(−16) and used the survival distribution described in Section 2.2.2.

For each simulated cancer patient, we calculated time to metastasis $t_{\text{met}}$ as

$$t_{\text{met}}=t_{\text{1s}}+t_{0}-t_{\text{det}}$$

where $t_{\text{1s}}$ is the time to the first seeding, $t_{0}$ is the time it takes for each tumour to growth from volume $V_{\text{cell}}$ to volume $V_{0}$ (conditional on each subject-specific inverse growth rate $r_{i}$ ), and $t_{\det}$ is the time from onset to detection. Note, in fact, that all three of these times are individual-specific. Finally, we applied administrative censoring after 20 years of follow-up.

### 3.3 Estimands

The estimands of interest are the parameters of the model for tumour growth and metastatic spread in the presence of screening: $\theta =\{\tau_{1},\tau_{2},\beta_{1},\beta_{2},\sigma ,\eta \}$ . In practice, we model $\tau_{1}$ , $\tau_{2}$ and σ on the log-scale and $\eta^{\prime }=-\log\;(\eta )$ .

### 3.4 Methods

We fitted our model (described in Section 2.4) to each independently simulated data set. Standard errors of the model parameters were computed by inverting the Hessian matrix at the optimum, which we calculated using Richardson extrapolation as implemented in the R package numDeriv.^24–26^

### 3.5 Performance measures and number of replications

We considered the following performance measures:

- Bias, for quantifying whether the estimation procedure can retrieve the true data-generating parameters, on average. We also report average and median estimated values of each estimand.
- Empirical and model-based standard errors, to quantify the precision of the point estimates.
- Coverage probability, quantifying the probability that a confidence interval contains the true value of each estimand.

Further details on each performance measure are described elsewhere^27^; for completeness, we also report their Monte Carlo standard errors which quantify the uncertainty in their estimation.

To estimate the key performance measure (bias) with a given precision, we first ran 10 replications of the simulation study. Allowing a Monte Carlo standard error for bias of 0.01, we would require $n_{\text{sim}}=\text{Var}/{\text{MCSE}}^{2}\approx 244$ repetitions of our full simulation study. This calculation was based on assuming Var=0.0244 , which was taken as the largest value among all empirical and model-based standard errors for $\hat{\theta }$ obtained from the above-mentioned 10 replications. To be conservative we rounded up the number of repetitions to the nearest 100, which yielded a total of $n_{\text{sim}}=300$ .

### 3.6 Software

This simulation study was coded and run using R version 4.0.2 and analysed using the rsimsum package.^21,28^The likelihood function was optimised using the optim function in R and the limited-memory modification of the Broyden-Fletcher-Goldfarb-Shanno (BFGS) quasi-Newton method.^29^

### 3.7 Results

There were no serious convergence issues with the estimation algorithm. Convergence was achieved for 293 (97.67%) of the 300 repetitions.

The performance measures of this simulation study are summarised in Table 2. The shape and rate parameters were estimated (on the log-scale) with small bias (0.0327 and 0.0416, respectively) and slight undercoverage (91.13% and 89.76%); empirical and model-based standard errors were close. Nevertheless, the average (across repetitions) estimated mean and variance of the Gamma distribution were 0.5509 and 0.1179 (compared to true values of 0.5556 and 0.1235). Furthermore, the estimated distributions of the inverse growth rates were very close to the distribution from which the data was generated. Supplemental Figure S1 depicts the estimated inverse growth rate distributions and the distribution of estimated median doubling time, across repetitions. The parameters of the screening sensitivity function $\beta_{1}$ and $\beta_{2}$ were estimated with some bias ( − 9.04% and − 5.13%, respectively). Consequently, coverage probability was poor at 15.02% for the intercept $\beta_{1}$ and 80.89% for $\beta_{2}$ . To study poor coverage, we report an additional performance measure named bias-eliminated coverage.^27^ This showed that poor coverage can be attributed to bias, as bias-eliminated coverage was otherwise good (Table 2). Despite the above-mentioned bias, the fitted sensitivity functions did not differ too much from that specified under our data-generating mechanism (Supplemental Figure S2). This is however not surprising, given the small number of tumours of very small size at detection. Empirical and model-based standard errors were in close agreement. Finally, −log(η) and log(σ) were estimated accurately with negligible bias, and good coverage probability; empirical and model-based standard errors were, once again, close.

**Table 2.** Performance measures for the simulation study with screening data; values are point estimates with Monte Carlo standard errors in brackets, where applicable.

Performance measure	$\log\;(\tau_{1})$	$\log\;(\tau_{2})$	$\beta_{1}$	$\beta_{2}$	log(σ)	−log(η)
True θ	0.916	1.504	−5.000	0.700	−16.000	8.500
Mean $\hat{\theta }$	0.949	1.546	−4.548	0.664	−16.016	8.510
Median $\hat{\theta }$	0.942	1.541	−4.538	0.663	−16.018	8.508
Bias	0.033 (0.004)	0.042 (0.005)	0.452 (0.008)	−0.036 (0.002)	−0.016 (0.002)	0.010 (0.005)
Empirical SE	0.075 (0.003)	0.081 (0.003)	0.138 (0.006)	0.033 (0.001)	0.042 (0.002)	0.077 (0.003)
Model SE	0.071 (0.000)	0.079 (0.000)	0.157 (0.001)	0.034 (0.000)	0.041 (0.000)	0.073 (0.000)
Relative % error in model SE	−5.418 (3.914)	−2.369 (4.039)	13.241 (4.698)	3.479 (4.300)	−2.151 (4.046)	−6.080 (3.887)
Coverage	0.911 (0.017)	0.898 (0.018)	0.150 (0.021)	0.809 (0.023)	0.939 (0.014)	0.925 (0.015)
Bias-eliminated coverage	0.949 (0.013)	0.956 (0.012)	0.969 (0.010)	0.942 (0.014)	0.945 (0.013)	0.918 (0.016)

Overall, the largest Monte Carlo error for bias was 0.0081 and, as expected, given the number of repetitions, was <0.01 .

## 4 An analysis of distant metastatic spread in Swedish post-menopausal breast cancer patients

We used our modelling approach to analyse data from a case-control study of postmenopausal breast cancer in Sweden (CAHRES; Cancer And Hormone REplacement Study).^30^ Data on the participants (women born and residing in Sweden, aged 50–74 years old, diagnosed with an incident primary invasive breast cancer between 1st October 1993, to 31st March 1995) was linked to data from the Swedish Cancer Registry and the Stockholm-Gotland Breast Cancer Registry. The data on distant metastases has been used before to study the association of mammographic density and the risk of distant spread.^31^ Mammographic images and screening histories were collected from mammography screening units and radiology departments, in an extension of the original case-control study; the collection of this data has also been described previously.^32,33^ The data analysed here is from 1614 breast cancer patients, of whom 1035 (64.13%) were detected via screening; the remaining 579 (35.87%) women detected their tumour symptomatically. Among all participants, the median tumour size at detection (as diameter) was 15 mm (inter-quartile interval [IQI]: 10–22 mm).

Four (0.25%) women had metastases at diagnosis; none of these women had small tumours (12, 28, 50 and 70 mm). From the women that were free of detected metastases at detection of the primary tumour, 293 (18.20%) were diagnosed with metastases during follow-up (Table 3). Median follow-up, estimated using the reverse Kaplan-Meier method,^34^ was 5.49 years (IQI: 5.41–5.58 years). As shown in Table 3, screen-detected tumours are, on average, smaller than symptomatic-detected tumours. The number of person-years at risk and events by tumour size quintiles and mode of detection are included in Table 3. Average rates of spread increase by tumour size and it appears that, taking tumours of similar size, there is more spread in symptomatic cases (which are, on average, faster-growing tumours) than in screen-detected tumours (although there are of course differences in tumour size distributions even within tumour size groups), which is consistent with our model (e.g. equation (19)).

**Table 3.** Summary of time at risk and number of events (detected metastases) by tumour size (quintiles: diameter, in mm) and mode of detection, for women free of metastases at detection of the primary tumour. Four women from the symptomatic cases group, i.e., those with detected metastases at diagnosis of the primary tumour, are excluded from this table. Event rates are depicted per 1000 person-years.

	Screen-detected cases				Symptomatic cases			
Tumour size (mm)	*N* (%)	Person-years	Events	Rate	*N* (%)	Person-years	Events	Rate
Q1 [1, 10]	380 (36.7%)	2020.76	19	9.40	78 (13.6%)	431.16	6	13.92
Q2 (10, 13]	160 (15.5%)	865.15	5	5.78	46 (8.0%)	252.91	4	15.82
Q3 (13, 18]	225 (21.7%)	1181.07	20	16.93	119 (20.7%)	571.63	30	52.48
Q4 (18, 25]	187 (18.1%)	903.56	44	48.70	161 (28.0%)	706.88	60	84.88
Q5 (25, 150]	83 (8.0%)	373.05	27	72.38	171 (29.7%)	646.69	78	120.61

We fitted our model (Section 2.4), first fixing k=4 (since this value of k has been shown to provide a good fit to data for a similar model for regional spread^10^). We also fitted the model assuming different integer values of k∈{1,2,3,5} ; log-likelihood values for each model with varying values of k are presented in Table 4. We can see that the model with k=4 has the highest maximum log-likelihood value. We even fitted a model that estimates k alongside the other model parameters. This yielded an estimated value of k=3.887 , with a log-likelihood value of −4559.10 . We, therefore, decided to proceed with the model with k=4 since integer values are attractive for interpretability. Parameter estimates for this model are given in Table 5.

**Table 4.** Log-likelihood values for models with different values of k .

k=1	k=2	k=3	k=4	k=5
−4669.44	−4596.21	−4567.58	−4559.18	−4565.68

**Table 5.** Model parameter estimates obtained from CAHRES data, assuming k=4 ; standard errors are estimated by inverting the Hessian matrix at the likelihood’s optimum.

	Estimate	SE	95% CI
$\log\;(\tau_{1})$	0.746	0.101	0.549 to 0.943
$\log\;(\tau_{2})$	0.910	0.162	0.592 to 1.227
$\beta_{1}$	−4.654	0.133	−4.914 to −4.394
$\beta_{2}$	0.439	0.022	0.396 to 0.483
−log(η)	9.078	0.081	8.920 to 9.237
log(σ)	−16.399	0.082	−16.559 to −16.239

Since examples of how continuous growth models can be used to estimate aspects of screening sensitivity and growth of the primary tumour (in CAHRES data) have been presented elsewhere,^8–10^ we focus here on novel quantities that we can obtain by modelling future events (distant metastases).

**Figure 2.**
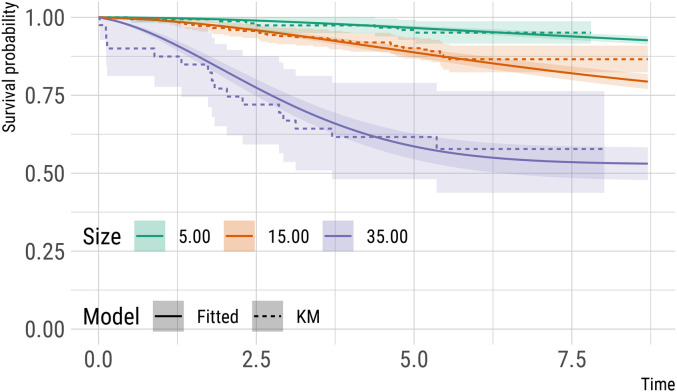
Estimates of survival (from distant metastases) probabilities (and 95% point-wise confidence intervals) for three hypothetical patients detected via screening, with a history of two negative screens, and with varying tumour size at detection. These model-based predictions assume no detected distant metastases at diagnosis and full removal of the primary tumour. Kaplan-Meier estimates for comparable subsets of subjects in CAHRES (see text) are included as a comparison.

The (hazard) rate of detection of first distant metastasis is defined in equation (15) as a function of σ , r , and (current) tumour volume. For our model with k=4 the point estimate of σ is exp(−16.399) (Table 5). In Figure 2 (solid line), we display model-based estimates, based on equation (14), over time, of the probability of detected distant metastatic spread for three hypothetical patients whose primary tumours were detected via screening and who had two previous negative screens, two years apart from each other. The three hypothetical patients differ only by the sizes of their tumours at diagnosis; as expected, patients with larger tumours have a shorter time to first detected distant metastasis. For instance, 5 years after diagnosis the probabilities of being free of distant metastasis for these three patients, with tumour sizes at detection of 5, 15 and 35 mm, are 0.97, 0.89 and 0.59, respectively. Standard errors for the model-based predictions were obtained using parametric bootstrap with 1000 bootstrap re-samples. For each hypothetical patient we selected a subset of patients from CAHRES with similar characteristics (i.e. detected via screening) and tumour sizes at detection of 2.50–7.50  mm (*n* = 161), 12.50–17.50  mm (*n* = 326) and 32.50–37.50  mm (n = 40), respectively, and plotted their Kaplan-Meier curve estimates (Figure 2, dashed line).

**Figure 3.**
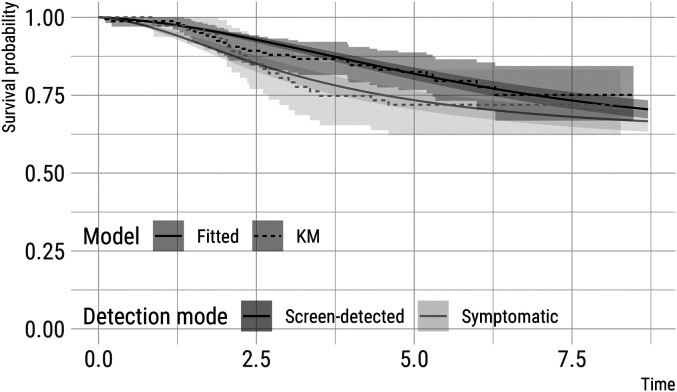
Estimates of survival (from distant metastases) probabilities (and 95% point-wise confidence intervals) for two hypothetical patients with a tumour of 20 mm at detection and a history of two negative screens. One subject is detected via screening, while the other subject is detected symptomatically six months after the previous negative screen. These model-based predictions assume no detected distant metastases at diagnosis and full removal of the primary tumour. Kaplan-Meier estimates for comparable subsets of subjects in CAHRES (see text) are included for comparison.

Figure 3 depicts survival probabilities for two hypothetical patients with a primary tumour of diameter 20 mm at detection and (once again) with a history of two previous negative screens, 2 years apart from each other. One subject is detected via screening, while the other subject is detected symptomatically 6 months after their previous negative screen: the subject detected symptomatically has a worse prognosis than the subject detected via screening. Again, we include standard errors for these model-based predictions, obtained using parametric bootstrap with 1000 bootstrap re-samples, and Kaplan-Meier curves (for each hypothetical patient) fitted on a comparable subset of patients from CAHRES (patients with tumour of size 17.50–22.50  mm and detected via screening, *n* = 159, or symptomatically with a negative screen in the last 2 years, *n* = 75).

**Figure 4.**
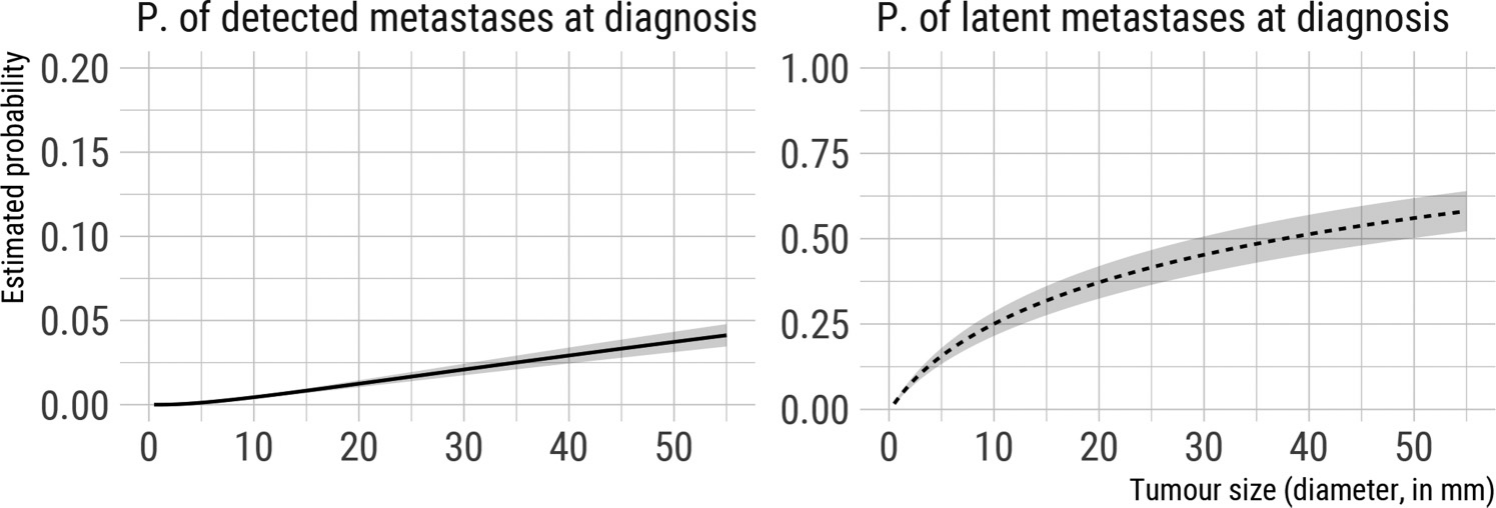
Estimated probability of latent and detected metastases at detection of the primary tumour (diagnosis) as a function of tumour size, based on model estimated on CAHRES data and assuming k=4 , with 95% point-wise confidence intervals.

Finally, based on our model, we estimated (1) the probability of a patient having dormant/latent metastases at diagnosis and (2) the probability of detecting distant metastases in a patient at diagnosis of the primary tumour, both as a function of tumour size at detection (Figure 4). Once again, standard errors are obtained using parametric bootstrap with 1000 bootstrap resamples. Averaging over the observed tumour size distribution in CAHRES, the estimated probability of having dormant/latent metastases at diagnosis was 0.327. Similarly, the marginal probability (based on the CAHRES tumour size distribution) of detecting distant metastases at diagnosis in CAHRES was calculated to be 0.011. These values are comparable to those that have been reported elsewhere.^35^

## 5 Robustness to misspecified model assumptions

In this section, we discuss the robustness of the proposed modelling framework to violations of some of the underlying, structural modelling assumptions; we assess this using Monte Carlo simulation. For studying robustness to misspecified assumptions it will not matter whether we focus on a screened or unscreened population. Therefore, for simplicity, we based our simulations on the implementation of our model in the absence of screening (Sections 2.2 and 2.3).

The assumptions that we assess (in turn) are the following:

- the primary tumour and seeded metastases grow at the same inverse growth rate r . This is assumption (1) in Section 2.2.2;
- Growth rates follow a Gamma distribution (equation (2));
- All individuals share the same spread parameter σ (equation (7)). Recall that the rate of spread is a function of σ and the individual, tumour-specific inverse growth rate r .

We relax (i) by simulating correlated growth rates from a bivariate Gaussian copula with equally distributed Gamma margins and a correlation parameter ρ ; we generated data under ρ values of 1.00 , 0.90 and 0.75 . We relax (ii) by simulating growth rates from a log-normal distribution. Finally, we relax (iii) by generating σ as a Gamma-distributed random variable. As a check on our simulation procedure, we also generated data under our original data-generating procedure; therefore, we generated data under six scenarios, four of which violated an assumption of our model. In all cases, we fitted the model described in Sections 2.2 and 2.3. Our simulation study is described in more detail in Appendix D in the Supplemental Material available online; we briefly summarise our results here.

We assessed the performance of the model when its assumptions are violated by comparing model-based predictions with empirical probabilities estimated on a large simulated dataset. We did this in terms of clinically relevant, model-based predictions; we assessed median tumour doubling times, probabilities of detected metastases and latent metastases at diagnosis of the primary tumours, fitted distributions of tumour volume at symptomatic detection, and conditional survival probabilities (for being free from detected distant metastases), conditional on being free from metastases and on having a particular tumour volume at diagnosis of the primary tumour. Here we present results only for the latter; results for the other prediction types are presented in Supplemental Material Appendix D.

**Figure 5.**
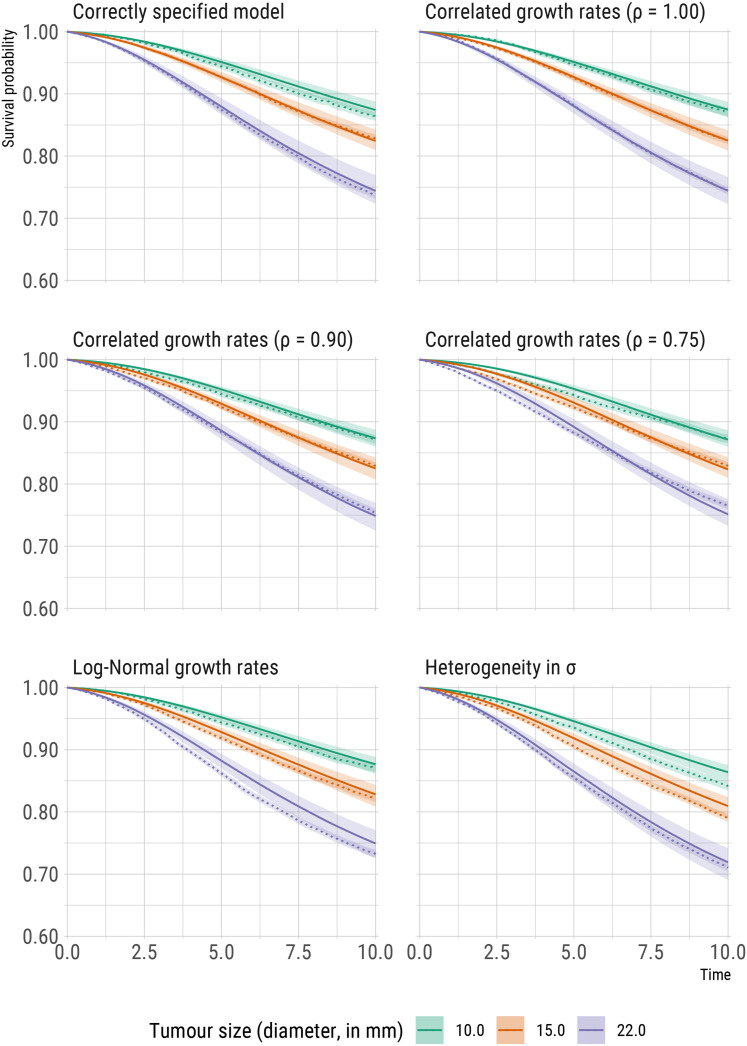
Comparisons of model predictions (solid lines) and reference, non-parametric estimates (dotted lines, using the Kaplan-Meier estimator) of time to diagnosis of distant metastasis (denoted in the plots as *survival probabilities*) for three distinct values of tumour size (diameter, in mm) at diagnosis of the primary tumour.

Predicted conditional survival probabilities (calculated using a procedure similar to that described in Section 2.5), under the six simulation scenarios and for three distinct values of tumour size at detection, are included in Figure 5. For comparison, Kaplan-Meier curves from the large simulated dataset are also included. Under all simulation scenarios, predicted survival probabilities do not dramatically differ from the observed, empirical probabilities and the effect of tumour size on the survival probabilities is preserved. Violations of assumptions are however reflected/detectable, in particular when (1) the correlation between growth rates of the primary tumour and metastases weakens, (2) the distribution of the growth rates is misspecified, and (3) we fail to account for heterogeneity in the spread parameter σ . In the next section, we discuss how our model can be extended to relax some of the assumptions addressed in the simulation scenarios.

## 6 Discussion

Continuous natural history growth models with random effects provide a useful alternative to multi-state (semi-) Markov models for studying the unobserved history of breast cancer. Models that have been introduced include those for tumour growth, time to symptomatic detection, screening sensitivity and local spread to the lymph nodes.^3–10^As far as we are aware, the model we introduce in this paper, that includes a component for events (distant metastases) that occur after the diagnosis of the primary tumour, is the first of its kind, although a similar model, but without random effects (that assumes that all primary tumours have the same rate of growth) has been proposed by Szczurek et al.^36^ Several models for events occurring after diagnosis have, of course, been developed, e.g., for survival after breast cancer surgery,^37,38^ breast cancer relapse,^39–43^ metastasis after breast conservation treatment,^44^ susceptibility to breast cancer,^45^ major adverse cardiovascular events in breast cancer patients.^46^However, these models condition on patients’ characteristics that are observed at diagnosis, i.e., do not incorporate the natural history of the disease. The model introduced here incorporates the unobserved natural history of the tumour. It relates the underlying biology to future outcomes, whilst at the same time providing insights into the latent growth and spread processes of the tumour. According to a recent review on prognostic models for breast cancer survival and recurrence, the most common model inputs are nodal status, tumour size, tumour grade, age at diagnosis, and oestrogen receptor status.^47^ In our model, we directly model tumour size and include it as a sole covariate in the screening sensitivity function, but it would be straightforward to accommodate other covariates in this and other parts of the model.

There exist several mathematical models for breast cancer distant metastasis, including models for the initiation of metastatic growth, and models to assess the efficacy of surgery and adjuvant therapy on metastatic spread (for a summary see Clare et al.^48^). Blumenson and Bross^49^ derived a model for the probability of a distant metastasis that is not removed by surgical treatment at diagnosis; they further extended their model to accommodate two distinct disease subgroups, with distinct model parameters. Slack et al. introduced another two-disease model describing the course of breast cancer and implications for clinical practice arising from the two-disease hypothesis.^50^ Jorcyk et al. described an animal model and a mathematical model for breast cancer progression, with the latter being solved numerically and showing good agreement with laboratory data.^51^ Models developed by Koscielny et al.^52^ and by Szczurek et al.^36^ are perhaps most relevant to our work. Koscielny et al. estimated that ∼ 30% of patients have metastases that are less than 12 months old at the time of initial diagnosis, based on a novel modelling approach. Equivalently, this suggests that the proportion of patients with metastases would be reduced by 30% if the primary tumours were treated 12 months earlier. Szczurek et al. proposed a stochastic model where the probability of a cell to successfully form a metastasis depends on the size of the newly formed colony; their approach is motivated by the empirical observation that cells in a growing colony benefit from a friendly environment when surrounded by more of their kind. Their approach enables the identification of a critical size that separates metastases that are very unlikely or very likely to survive, which they named *metastatic bottleneck*. As mentioned above, a major limitation of Szczurek et al.’s model is that it assumes a common rate of tumour growth (and therefore spread rate). In our work we assume that it can vary between individuals; previous estimates of tumour growth rate variation have shown substantial heterogeneity between subjects.^6^

The model we describe in this paper has, of course, its limitations. It relies on a set of assumptions (described in Section 2 and summarised in Table 1), among which that detection of the primary tumour is a function only of its size (i.e. it is independent of the process of metastatic spread), that metastases growing to a detectable size before the diagnosis of the primary tumour are only visible at diagnosis of the primary tumour, that metastatic seeding completely stops at diagnosis of the primary tumour, and that already seeded, successful colonies are not affected. The first two of these exclude the possibility for *cancers of unknown primary*. The third and fourth are concerned with the efficiency of surgery and therapy in eradicating tumour from the breast and regional lymph nodes, and in preventing metastatic recurrence. Data analysed here is from a study carried out on women diagnosed before the introduction of, e.g., ERBB2-targeted therapy.^53^ Even still, our assumptions will not be completely realistic, e.g., we do not incorporate the effects of chemotherapy, which, depending on the drug, aim to disrupt DNA replication or mitosis. Ideally, we would like to be able to explore approaches for modelling the efficacy of treatments at diagnosis.^54,55^ Metastatic seeding is unlikely to completely stop when the primary tumour is removed surgically, even when adjuvant therapy is administered.^56^Incorporating and modelling the complexities of breast cancer treatments and cancer subtype differences is a natural extension of our model. Rather than assuming that all *successful* metastases are not affected by therapy, with sufficient data, it would be possible to relax the assumption by, e.g., allowing a proportion of seeded metastases to be eradicated or for growth to be slowed. However, in practice, the model we propose will be of use and may be reasonable since, as mentioned in the introduction, there is strong evidence (e.g. from studies looking at molecular mechanisms) that most metastases are seeded early.^20^ There are of course even other assumptions implicit in our approach, that are not mentioned above. In reality, seeding of cancer metastases may in some cases be complex.^57^ For example, implicitly we assume that metastases do not cross-seed new metastases; cross-seeding may contribute to faster, or increase heterogeneity in, metastatic growth.

We examined the robustness of our model to misspecification of some of the structural assumptions, using simulation, in Section 5: key patterns in the data were captured. We note that some of the assumptions that we relaxed could be incorporated into our model without too much difficulty. It would, for example, be straightforward to include a random effect for the spread parameter σ . This would involve replacing the density in equation (12) by a marginal distribution integrating over the random effect distribution; the likelihood contribution could subsequently be easily modified. Instead of assuming that (given the growth rate of the primary tumour) the size/time at which distant metastases are detected is fixed, corresponding to volume $V_{0}$ , it would also be relatively straightforward to estimate this volume or even to allow the growth rate of the distant metastases to differ from the growth rates of the primary tumour by a fixed (multiplicative) factor. It would even be possible to allow the growth rates of the primary tumour and the distant metastases to be imperfectly correlated by specifying a bivariate distribution. The density of W would then require evaluating a double integral instead of a single integral (see Section 2.3), which would increase computation time. This extension would be interesting to investigate: whilst there is some evidence supporting that growth rates and proliferation indices of paired metastases and primary tumours are similar,^58–60^ they are not likely to be identical. In future work, we will address some of the model extensions mentioned above.

The conventional, sequential model of breast cancer progression states that cancer passes through several stages disseminating first to the lymph nodes and then to distant organs. However, genomic analyses suggest that there is minimal seeding from axillary lymph nodes.^61^ Our current model for distant metastatic spread does not include a component for local breast cancer spread to the lymph nodes (e.g. as described in the work of Isheden and Humphreys^10^). In future work, we plan to incorporate both components to fit with the conventional model of breast cancer progression.

Even in its current form, our model adapts to biological principles of breast cancer growth and spread providing building blocks for future extensions that could accommodate more complexity and processes within a single, unified and efficient framework for modelling latent tumour growth and future outcomes. Models and, more importantly, frameworks of this kind are fundamentally important for studying novel interventions (such as personalised screening) and treatments, e.g., using microsimulation, or for studying risk factors behind tumour growth and spread.

## Supplemental Material

sj-pdf-2-smm-10.1177_09622802211072496 - Supplemental material for Estimating latent, dynamic processes of breast cancer tumour growth and distant metastatic spread from mammography screening data Click here for additional data file. Supplemental material, sj-pdf-2-smm-10.1177_09622802211072496 for Estimating latent, dynamic processes of breast cancer tumour growth and distant metastatic spread from mammography screening data by Alessandro Gasparini and Keith Humphreys in Statistical Methods in Medical Research

sj-pdf-3-smm-10.1177_09622802211072496 - Supplemental material for Estimating latent, dynamic processes of breast cancer tumour growth and distant metastatic spread from mammography screening data Click here for additional data file. Supplemental material, sj-pdf-3-smm-10.1177_09622802211072496 for Estimating latent, dynamic processes of breast cancer tumour growth and distant metastatic spread from mammography screening data by Alessandro Gasparini and Keith Humphreys in Statistical Methods in Medical Research
