# Supplementary material for: Estimating latent, dynamic processes of breast cancer tumour growth and distant metastatic spread from mammography screening data
Source: Stat Methods Med Res. 2022 Feb 1;31(5):862–81. doi: 10.1177/09622802211072496 (PMC9099158; doi:10.1177/09622802211072496)
Supplement: sj-pdf-3-smm-10.1177_09622802211072496 - Supplemental material for Estimating latent, dynamic processes of breast cancer tumour growth and distant metastatic spread from mammography screening data [file sj-pdf-3-smm-10.1177_09622802211072496.pdf]

# Supplementary Material: *Estimating Latent, Dynamic Processes of Breast Cancer Tumour Growth and Distant Metastatic Spread from Observational Screening Data*

Alessandro Gasparini and Keith Humphreys

2021-11-17

## A Simulation Study for the Model in the Absence of Screening

### A.1 Aim

We designed a simulation study to assess the ability to retrieve the true data-generating parameters of the model for data on tumour size and time to first distant metastasis, for a sample of incident cases collected in the absence of screening.

### A.2 Data-Generating Mechanisms

We simulated independent datasets of 1500 individuals each, assuming tumours originate from a single spherical cell of volume $V_{\text{cell}}$ corresponding to a diameter of $d_{\text{Cell}} = 0.01$ mm; we also assumed that tumours are detectable only when when they reach a given volume $V_0$ corresponding to a diameter $d_0 = 0.5$ mm. We assumed the parameter $k$ to be fixed at $k = 4$.

We simulated inverse growth rates from a GammaF distribution with equal shape and rate parameters $\tau_1 = \tau_2 = \tau$ (for identifiability purposes), and we fixed $\tau = \exp(0.2938482)$. This corresponds to a GammaF distribution with a mean of 1 and a variance of approximately 0.75).

Then, we simulated the volume of the tumour at symptomatic detection at the time from inception to detection from the model for the rate of symptomatic detection and the density for tumour volume at symptomatic detection; we fixed the parameter $\eta' = -\log(\eta) = 9.3811683$.

The number of metastases being seeded per each simulated individual was drawn from a Poisson distribution with subject-specific intensity depending on $t = t_{\text{det}}$ (simulated at the previous step) and assuming $\sigma = \exp(-15.5223994)$. The timing of the first seeding was simulated using the inversion method as described in Bender et al. (2005).

The true values of the parameters described above were selected by fitting the model to a subset of data from CAHRES consisting of women that were detected symptomatically and with no history of previous negative screens. CAHRES is described in more detail in the body of the manuscript and elsewhere (Eriksson et al. 2013; Rosenberg et al. 2006, 2008).

We therefore calculated time to metastasis $t_{\text{met}}$ as the sum

$$t_{\text{met}} = t_{1s} + t_0 - t_{\text{det}},$$

where $t_{1s}$ is the time to the first seeding, $t_0$ is the time it takes for each tumour to growth from volume $V_{\text{Cell}}$ to volume $V_0$ (conditional on each subject-specific inverse growth rate $r_i$), and $t_{\text{det}}$ is the time to symptomatic

detection (simulated above).

Finally, we applied administrative censoring after 20 years of follow-up.

## A.3 Estimands

The estimands of interest are the parameters of the model for tumour growth and metastatic spread in the absence of screening: $\theta = \{\tau, \sigma, \eta\}$. In practice, we modelled $\tau$ and $\sigma$ on the log-scale and $\eta' = -\log(\eta)$.

## A.4 Methods

We fitted the true data-generating model in the absence of screening (as described in the body of the manuscript) to each independent simulated dataset. Standard errors of the model parameters were computed by inverting the Hessian matrix at the optimum, which was calculated using Richardson extrapolation as implemented in the R package `numDeriv` (Fornberg and Sloan 1994; Gilbert and Varadhan 2019; Lindfield and Penny 1989).

## A.5 Performance Measures and Number of Replications

Given the aim of this simulation study, the performance measures of interest are:

- Bias, to quantify whether the estimation procedure can retrieve the true data-generating parameters on average. This is the key performance measure. We also report average and median estimated values of each estimand;
- Empirical and model-based standard errors, to quantify the precision of the point estimates;
- Coverage probability, quantifying the probability that a confidence interval contains the true value of each estimand.

Further details on each performance measure are described elsewhere (Morris et al. 2019); we report Monte Carlo standard errors of each performance measure as well, quantifying the uncertainty in their estimation.

To estimate the key performance measure with a given precision, we first ran 10 replications of this simulation study. Allowing a Monte Carlo standard error for bias of 0.01, we would require $n_{\text{sim}} = \text{Var}/\text{MCSE}^2 \approx 31$, where $\text{Var} = 0.0031$ is estimated from the largest value among all empirical and model-based standard errors for $\widehat{\theta}$ obtained from the above-mentioned 10 replications. To be conservative we rounded up that value to the nearest 100 (with a minimum of 200 replications), yielding a total of $n_{\text{sim}} = 200$.

## A.6 Software

This simulation study was coded and run using R version 4.0.2 and analysed using the `rsimsum` package (Gasparini 2018; R Core Team 2020). The likelihood function was optimised using the `optim` function in R and the limited-memory modification of the Broyden-Fletcher-Goldfarb-Shanno (BFGS) quasi-Newton method (Byrd et al. 1995).

## A.7 Results

Descriptive characteristics of the metastatic process across all simulated datasets are presented in Table S1. On average, 5.07% of individuals had detectable metastases at the time of primary tumour detection, 64.04% developed a detectable metastasis during follow-up, and 30.89% were event-free at the end of follow-up. The proportion of individuals developing detectable metastases during follow-up was rather high, but we did not simulate any competing event (such as death) here.

All models converged to a solution, with no replications showing issues with the convergence of the optimisation algorithm or calculation of the Hessian matrix.

Table S1: Summary of descriptive characteristics of metastatic process across simulated datasets.

| | Min | Q1 | Mean | Median | Q3 | Max |
|---|---|---|---|---|---|---|
| Pr. of metastases at detection | 0.0307 | 0.0460 | 0.0507 | 0.0513 | 0.0547 | 0.0693 |
| Pr. of events during follow-up | 0.6073 | 0.6320 | 0.6404 | 0.6400 | 0.6480 | 0.6727 |
| Pr. of event-free subjects | 0.2820 | 0.3017 | 0.3089 | 0.3090 | 0.3163 | 0.3373 |

Table S2: Performance measures of the simulation study; values are point estimates (and Monte Carlo standard errors, when applicable). True values of the estimands (used to generate data) are printed up to 4 significant digits.

| Performance Measure | $\log(\tau)$ | $-\log(\eta)$ | $\log(\sigma)$ |
|---|---|---|---|
| Actual $\theta$ | 0.2938 | 9.3812 | -15.5224 |
| Mean $\hat{\theta}$ | 0.3010 | 9.3832 | -15.5208 |
| Median $\hat{\theta}$ | 0.2995 | 9.3864 | -15.5225 |
| Bias | 0.0072 (0.0030) | 0.0020 (0.0024) | 0.0016 (0.0022) |
| Empirical SE | 0.0420 (0.0021) | 0.0342 (0.0017) | 0.0312 (0.0016) |
| Model SE | 0.0454 (0.0001) | 0.0336 (0.0000) | 0.0341 (0.0000) |
| Relative % Error in Model SE | 7.9250 (5.4045) | -1.6866 (4.9222) | 9.1614 (5.4651) |
| Coverage | 0.9650 (0.0130) | 0.9350 (0.0174) | 0.9750 (0.0110) |

The performance measures of this simulation study are summarised in Table S2. The average and median point estimates were very close to the true value of each estimand; most importantly, bias was negligible – ranging between 0.0016 and 0.0072 in absolute values (-0.01% to 2.44% in relative values). Monte Carlo errors for bias were $< 0.01$, as expected with the current number of replications.

The comparison of model-based and empirical standard errors showed that our procedure (inverting the numerical Hessian at the optimum) slightly overestimated the standard errors for $\log(\tau)$ and $\log(\sigma)$, with a relative percentage error of 7.93% and 9.16%, respectively; model-based standard errors for $-\log(\eta)$ were underestimated, with a relative percentage error of -1.69%.

Finally, coverage probability was good overall, between 93.50% and 97.50%.

# B Supplementary Figure S1

Supplementary Figure S1 compares the fitted distributions of inverse tumour growth rates $r$ with the distribution specified under the data-generating mechanism. Panel A includes each fitted distribution (in grey), across all repetitions, while panel B depicts the distribution of estimated median tumour doubling times, again, across repetitions. In the plots, the red line/dot represents the distribution/value from which the data was generated.

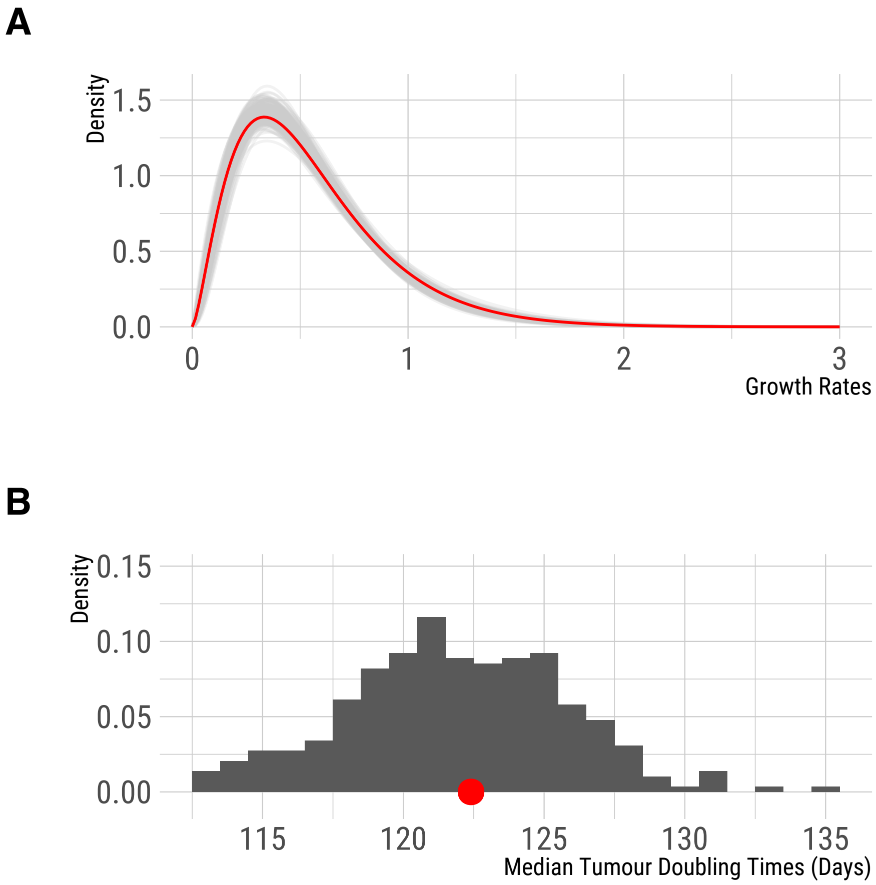


Figure S1: Distribution of inverse growth rates and median doubling times across simulation repetitions.

# C Supplementary Figure S2

Supplementary Figure S2 compares fitted screening sensitivity functions (for the simulation study in the presence of screening) to that specified under the data-generating mechanism. The grey shaded area identifies the range of fitted sensitivity curves across all repetitions of the simulation study as described in the body of the manuscript.

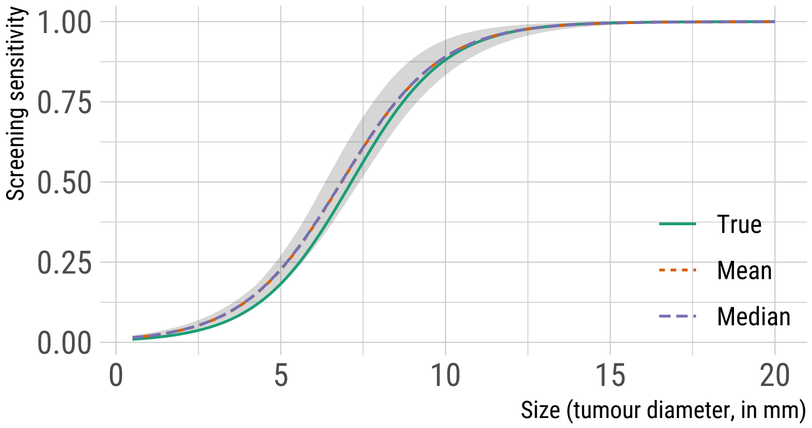


Figure S2: Screening sensitivity functions across simulation repetitions.

# D Simulation Study to Examine Robustness to Model Misspecification

## D.1 Aim

This simulation study aims to assess the robustness of the proposed model to alternative data-generating mechanisms. We focus on the model in the absence of screening, for (1) simplicity and (2) to keep the focus on the core of the new model introduced in this paper.

## D.2 Data-Generating Mechanisms

We simulate data under five different data-generating mechanisms. Specifically, we define three scenarios where we relax the assumption of common growth rates between the primary tumour and seeded metastases (scenarios 1-3), where we relax the assumption of Gamma-distributed growth rates (scenario 4), and where we allow for heterogeneity in the spread parameter $\sigma$ (scenario 5).

In scenarios 1-3, we simulate growth rates for the primary tumour and each seeded metastasis from a bivariate distribution that we constructed using a bivariate Gaussian copula (Meyer 2013) with correlation parameter values $\rho = 1.00, 0.90$, and 0.75, respectively. The margins of the two growth rate distributions are assumed to be Gamma-distributed, with equal shape and rate parameters $\tau_1 = \tau_2 = 2.3$. Note that scenario 1 corresponds to perfectly correlated growth rates, and therefore represents the model introduced in the main body of the paper; it was however included as a check on the data generation procedure based on the copula model.

In scenario 4, we relax the assumption that growth rates follow a Gamma distribution. Specifically, we simulate growth rates from a log-Normal distribution with mean and standard deviation (on the log-scale) of $-0.25$ and 0.75, respectively.

In scenario 5 we allow for heterogeneity in the spread parameter $\sigma$. Specifically, we draw subject-specific values of the spread parameter from a Gamma distribution with scale and rate parameters of $e^0$ and $e^{16}$, respectively.

For all scenarios, we simulate data assuming $\eta' = 9.0$; the remaining parameter values and assumptions are the same as those used in the simulation study of Appendix A.

## D.3 Estimands

We consider four model-based predictions under each data-generating scenario:

1. Median tumour doubling time (in days), estimated as

$$\text{median(of the fitted Gamma distribution)} \times \log(2) \times 365.242$$

2. Conditional probability of having detected metastases at diagnosis of the primary tumour (given tumour volume $v_{\text{det}}$), estimated as

$$P(\text{Detected metastasis at diagnosis}) = 1 - \exp\left[-\hat{\sigma}\left(\log\frac{v_{\text{det}}}{V_0}\right)^{k+1}\right]$$

   with $\hat{\sigma}$ the coefficient estimated from the model. We estimate this quantity across a range of values for $v_{\text{det}}$;

3. Conditional probability of having undetected latent metastases at diagnosis, estimated as

$$P(\text{Latent metastasis at diagnosis}) = 1 - \exp\left[-\hat{\sigma}\left(\log\frac{v_{\text{det}}}{V_{\text{cell}}}\right)^{k+1}\right]$$

   Again, $\hat{\sigma}$ is estimated from the model and we obtain predictions across a range of values for $v_{\text{det}}$;

4. Predicted survival probability at time $w^*$ for subjects without detected metastases at diagnosis of the primary tumour. This is estimated as:

$$\int_0^{+\infty} \frac{P(W > w^*|V_{\text{det}} = v_{\text{det}}, R = r)}{P(W > 0|V_{\text{det}} = v_{\text{det}}, R = r)} f_R(r|V_{\text{det}} = v_{\text{det}})\, dr,$$

   where $P(W > w^*|V_{\text{det},R})$, $P(W > 0|V_{\text{det},R})$ are calculated based on Equation 16 in the manuscript. We obtain predictions across values of $w^*$ and for three distinct values of $v_{\text{det}}$ (corresponding to diameters of 10, 15, and 22 mm).

Finally, we consider the fitted distribution of tumour volume at symptomatic detection (i.e. Equation 4 in the manuscript).

These estimands were chosen to compare the potential effect of misspecifying the underlying biological mechanism on some of the clinically relevant quantities and predictions that could be obtained from the model described in this manuscript. Predictions are obtained after fitting the model to each simulated dataset and under each distinct scenario.

## D.4 Performance Measures

Predictions under each simulation scenario are presented/evaluated graphically. Specifically:

1. We compare the distribution of predicted median tumour doubling times using box plots;
2. We compare the probabilities of detected and latent metastasis at diagnosis (i.e. estimands 2 and 3, above) with empirical probabilities calculated from a large simulated dataset (with 100,000 subjects) under each

data-generating mechanism. Empirical probabilities are computed across categories of tumour sizes, defined as 5 mm wide intervals. Exact confidence intervals for the empirical probabilities are additionally included;

3. We compare the predicted survival probabilities to survival curves obtained using the Kaplan-Meier estimator on the same large simulated datasets used in point 2, above, with size categories defined using ±2.5 mm from the central point;

4. We compare the fitted distribution of tumour volume at detection with a histogram of tumour volumes on raw data;

5. Finally, to provide a comprehensive view of the results, we also include box plots for the distribution of fitted model parameters across scenarios and repetitions.

Furthermore, as an additional comparison, we include another scenario where we simulate data from the correct model (as in Appendix A) but using the parameters described in Appendix D.2 instead. This is referred to below as the *Reference*.

We run 200 repetitions per simulation scenario (as in Appendix A).

## D.5 Software

This simulation study was coded and run using R version 4.0.4; once again, the model likelihood was optimised using the `optim` function and the limited-memory modification of the BFGS algorithm (Byrd et al. 1995; R Core Team 2020).

## D.6 Results

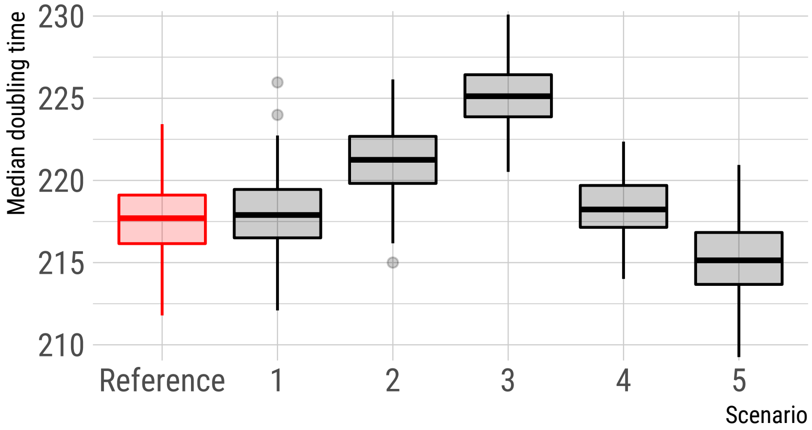


Figure S3: Distributions of median doubling times of primary tumours across simulation scenarios and repetitions. The *Reference* scenario is highlighted in red.

The distribution of median tumour doubling times across scenarios is depicted in Figure S3. The distributions largely overlap, with increasingly larger values in scenarios 2 and 3 as the correlation between growth rates of the primary tumour and metastases diminishes. Conversely, in the scenario with heterogeneity in the spread parameter, the median of the distribution was slightly lower. Nevertheless, differences between all scenarios are still relatively small (with a range of approximately 10 days between the largest and smallest median of each distribution).

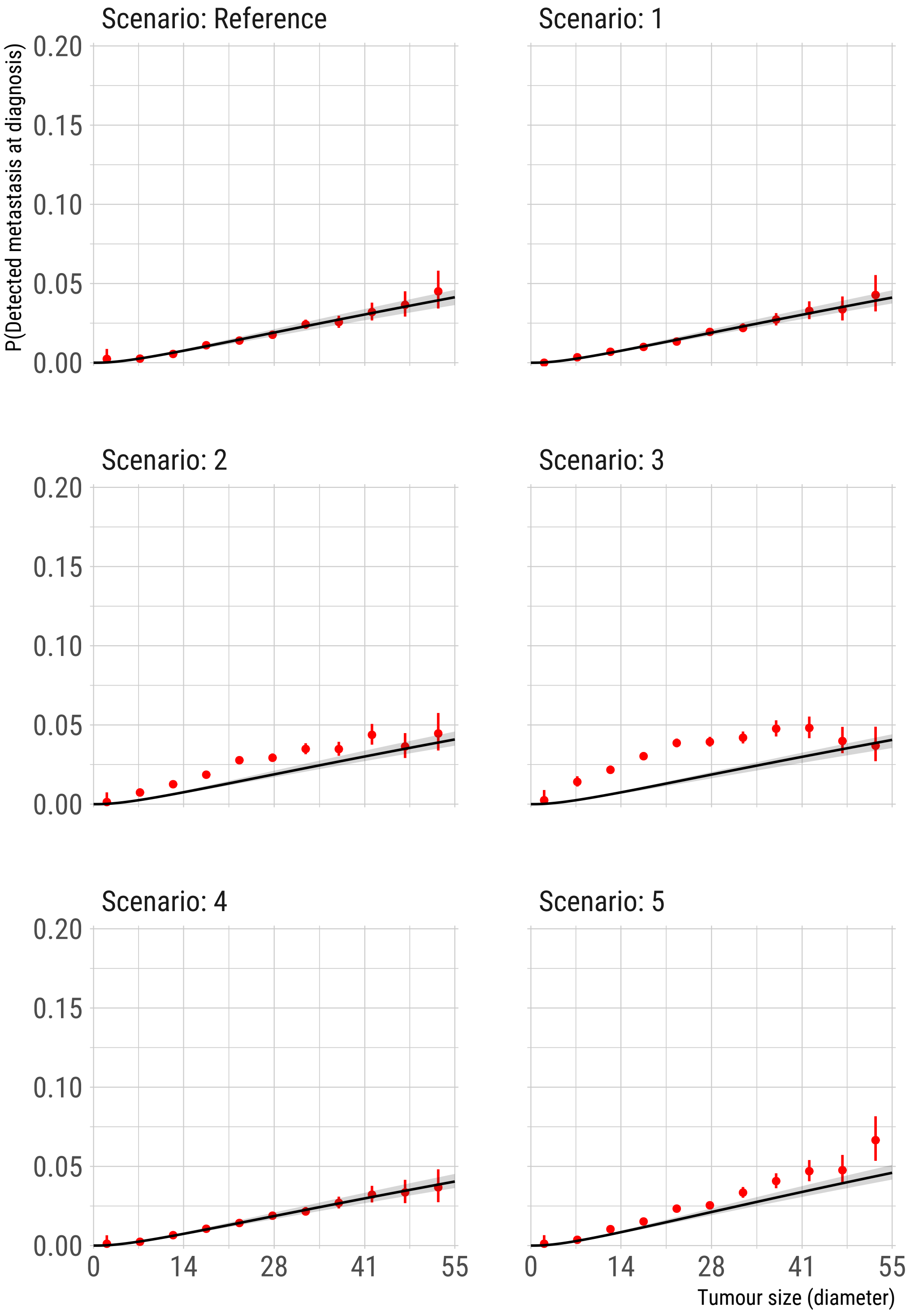


Figure S4: Model-based estimates of the probability of detected metastases at diagnosis. The solid black line represents the median prediction across all repetitions, with the shaded gray area representing the range (minimum to maximum) of predictions. For comparison, empirical probabilities calculated from a single large simulated dataset of 100,000 individuals are included in red.

Figure S4 depicts the fitted probabilities of detected metastases at diagnosis as a function of tumour size; as a comparison, we include empirical probabilities estimated on a single simulated (large) dataset. Under scenarios 1 and 4 predictions closely match the empirical probabilities. Under scenarios 2, 3, and 5, predictions based on the fitted model slightly underestimated the probabilities at some tumour sizes. This was most serious for scenario 3, where growth rates of the primary tumour and distant metastases were not strongly correlated.

Figure S5 depicts the fitted probability of having latent, yet undetected metastases at diagnosis as a function of tumour size; once again, empirical probabilities are included for comparison. All scenarios showed a decent fit between predicted and empirical probabilities, with the model only slightly overestimating the probability of having latent metastases.

Figure 4 in the main manuscript depicts survival (from detection of distant metastases) probability predictions over time for three distinct values of tumour size, conditional on no detected metastases at diagnosis of the primary tumour. We compare these predictions to Kaplan-Meier curves fitted to data from comparable sub-groups in the single simulated (large) dataset. Overall, considering the violation of model assumptions in each of the simulation scenarios, the predicted probabilities match the non-parametric Kaplan-Meier estimates reasonably well, although violations of assumptions are reflected/detectable in particular for scenarios 3 to 5 (where the fitted models slightly overestimated survival from metastases times).

Figure S6 compares the fitted and empirical distributions of tumour volume at symptomatic diagnosis of the primary tumour. Overall, the fit was good (across all simulation scenarios), indicating that the model misspecifications we considered, perhaps not surprisingly, show up mainly in the diagnostics related to distant metastases.

Finally, in Figure S7 we compare the parameter estimates across the simulation scenarios. Generating data based on non-perfectly-correlated growth rates between the primary tumour and metastases (scenarios 2 and 3) resulted in $\log(\tau_1)$ and $-\log(\eta)$ being (on average) overestimated, with there being increasingly larger differences as the correlation diminished. This corresponds to overestimating the median inverse growth rate (which is in accordance with Figure S3) and underestimating the rate of symptomatic detection. Conversely, $\log(\sigma)$ was estimated with little bias, across all scenarios, except for scenario 5 (data-generating mechanism with heterogeneity in $\sigma$) where $\log(\sigma)$ was slightly overestimated. Nevertheless, the bias was marginal and (as seen in the previous plots) did not substantially affect model-based predictions.

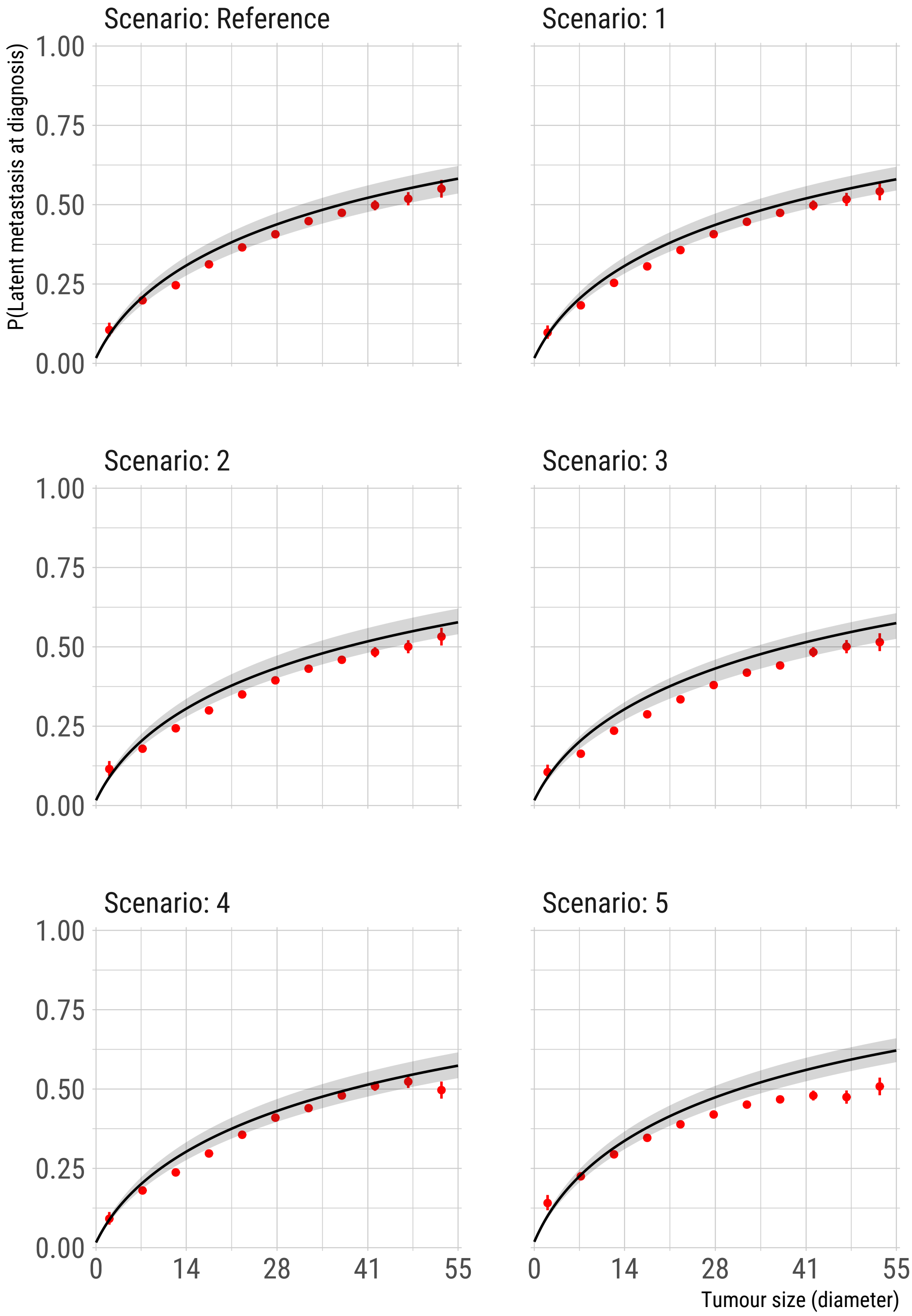


Figure S5: Model-based estimates of the probability of latent metastasis at diagnoses. The solid black line represents the median prediction across all repetitions, with the shaded gray area representing the range (minimum to maximum) of predictions. For comparison, empirical probabilities calculated from a single large simulated dataset of 100,000 individuals are included in red.

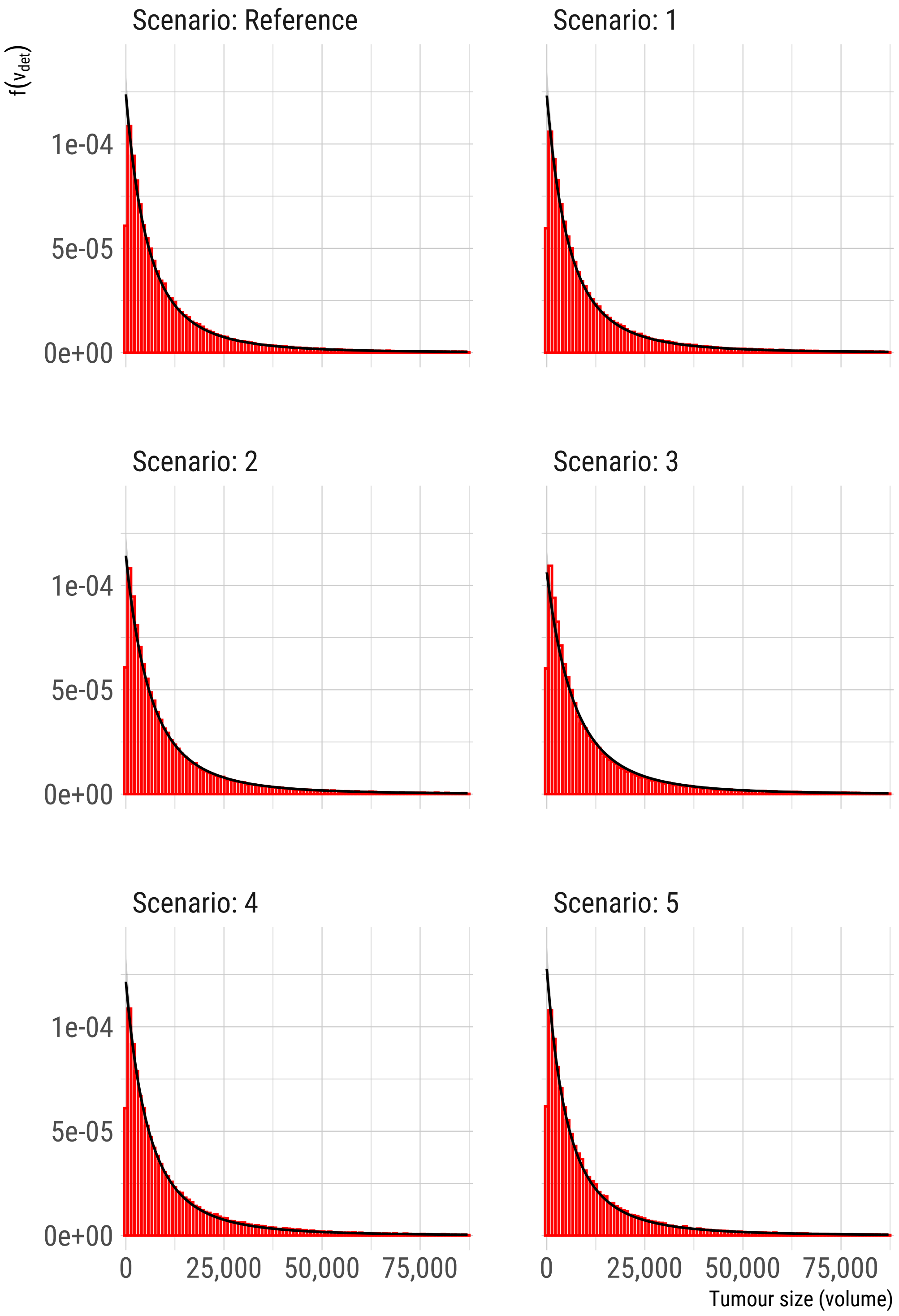


Figure S6: Fitted densities of tumour volumes at symptomatic diagnosis. The solid black line represents the median fitted value, with the grey shaded area representing the range of minimum to maximum predictions. For comparison, histograms of tumour volumes at diagnosis from a single simulated dataset with 100,000 individuals are included in red.

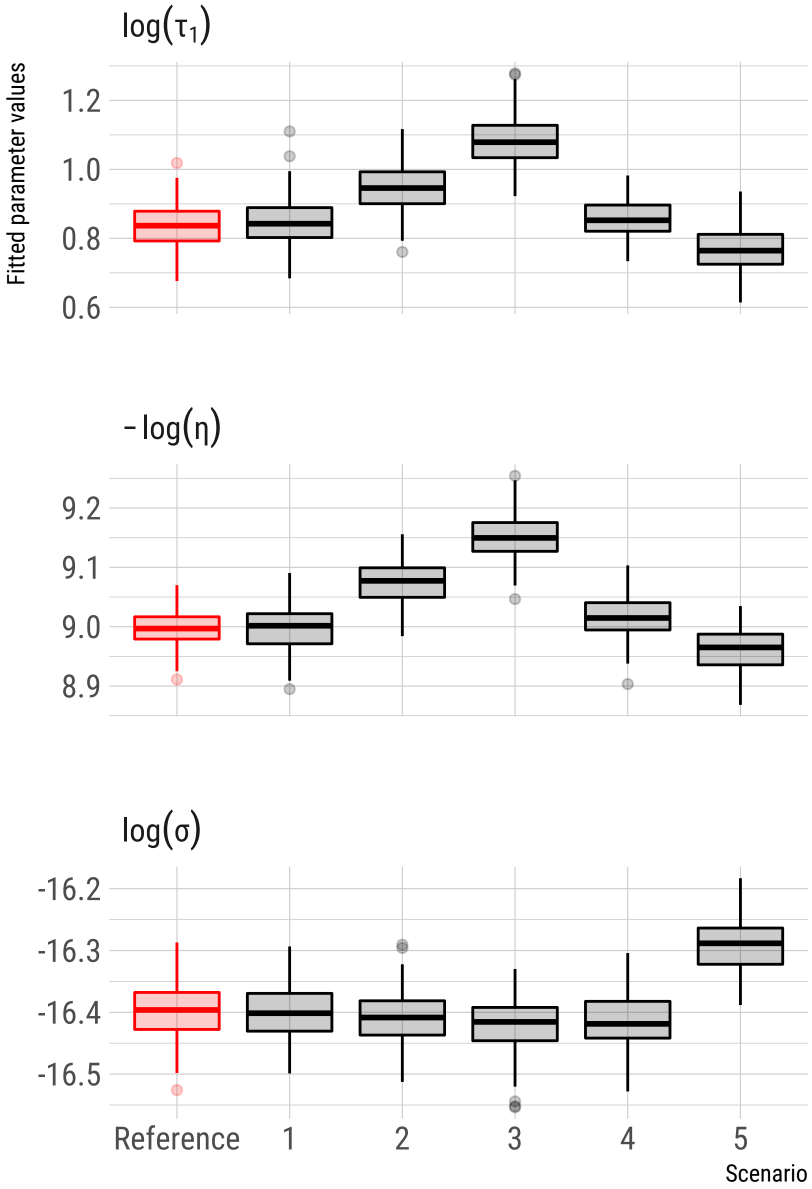


Figure S7: Box plots representing the distributions of fitted parameter values across simulation scenarios.

# Bibliography


Bender, R., Augustin, T., and Blettner, M. (2005), "Generating survival times to simulate cox proportional hazards models," *Statistics in Medicine*, 24, 1713–1723. https://doi.org/10.1002/sim.2059.

Byrd, R. H., Lu, P., Nocedal, J., and Zhu, C. (1995), "A limited memory algorithm for bound constrained optimization," *SIAM Journal on Scientific Computing*, 16, 1190–1208. https://doi.org/10.1137/0916069.

Eriksson, L., Czene, K., Rosenberg, L., Humphreys, K., and Hall, P. (2013), "Possible influence of mammographic density on local and locoregional recurrence of breast cancer," *Breast Cancer Research*, 15. https://doi.org/10.1186/bcr3450.

Fornberg, B., and Sloan, D. M. (1994), "A review of pseudospectral methods for solving partial differential equations," *Acta Numerica*, 3, 203–267. https://doi.org/10.1017/s0962492900002440.

Gasparini, A. (2018), "rsimsum: Summarise results from Monte Carlo simulation studies," *Journal of Open Source Software*, 3, 739. https://doi.org/10.21105/joss.00739.

Gilbert, P., and Varadhan, R. (2019), *numDeriv: Accurate numerical derivatives.*

Lindfield, G. R., and Penny, J. E. T. (1989), *Microcomputers in numerical analysis*, USA: Halsted Press.

Meyer, C. (2013), "The bivariate normal copula," *Communications in Statistics - Theory and Methods*, 42, 2402–2422. https://doi.org/10.1080/03610926.2011.611316.

Morris, T. P., White, I. R., and Crowther, M. J. (2019), "Using simulation studies to evaluate statistical methods," *Statistics in Medicine*, 38, 2074–2102. https://doi.org/10.1002/sim.8086.

R Core Team (2020), *R: A language and environment for statistical computing*, Vienna, Austria: R Foundation for Statistical Computing.

Rosenberg, L. U., Granath, F., Dickman, P. W., Einarsdóttir, K., Wedrén, S., Persson, I., and Hall, P. (2008), "Menopausal hormone therapy in relation to breast cancer characteristics and prognosis: A cohort study," *Breast Cancer Research*, 10. https://doi.org/10.1186/bcr2145.

Rosenberg, L. U., Magnusson, C., Lindström, E., Wedrén, S., Hall, P., and Dickman, P. W. (2006), "Menopausal hormone therapy and other breast cancer risk factors in relation to the risk of different histological subtypes of breast cancer: A case-control study," *Breast Cancer Research*, 8. https://doi.org/10.1186/bcr1378.
